# Supplementary material for: Spatially aware dimension reduction for spatial transcriptomics
Source: Nat Commun. 2022 Nov 23;13:7203. doi: 10.1038/s41467-022-34879-1 (PMC9684472; doi:10.1038/s41467-022-34879-1)
Supplement: Supplementary file 1 — Supplementary Information [file 41467_2022_34879_MOESM1_ESM.pdf]

Supplementary Information

Spatially Aware Dimension Reduction for Spatial Transcriptomics

Lulu Shang[1, 2], Xiang Zhou[1, 2#]

1. Department of Biostatistics, University of Michigan, Ann Arbor, MI 48109, USA

2. Center for Statistical Genetics, University of Michigan, Ann Arbor, MI 48109, USA

#Correspondence to XZ (xzhousph@umich.edu).

This PDF file includes:
Supplementary Note 1
Supplementary Figures 1-43
Supplementary Tables 1-13
Supplementary References

Supplementary Note 1

Algorithm for SpatialPCA

We describe the detailed algorithm for SpatialPCA. Specifically, we first integrate out both $\boldsymbol{B}$ and $\boldsymbol{Z}$ to obtain a marginal likelihood, based on which we infer τ, σ_0^2 and $\boldsymbol{W}$. We then estimate $\boldsymbol{Z}$ by computing their posterior mean conditional on the estimated τ, σ_0^2 and $\boldsymbol{W}$.

We first integrate out $\boldsymbol{B}$. To simplify notation, we denote $\boldsymbol{M} = \boldsymbol{I}_n - \boldsymbol{X}(\boldsymbol{X}^T\boldsymbol{X})^{-1}\boldsymbol{X}^T$ and $\boldsymbol{Y}^* = (\boldsymbol{Y} - \boldsymbol{W}\boldsymbol{Z})^T$, where we have $Y_i^* \sim MVN(X_i\boldsymbol{B}, \sigma_0^2\boldsymbol{I}_n)$. The marginal distribution for $\boldsymbol{Y}^*$ after integrating out $\boldsymbol{B}$ is:

$$
\begin{aligned}
p(\boldsymbol{Y}^* \mid \sigma_0^2) &= \int_{\boldsymbol{B}} L(\boldsymbol{B}, \sigma_0^2) d\boldsymbol{B} = \int_{\boldsymbol{B}} \frac{1}{(2\pi\sigma_0^2)^{\frac{n}{2}}} e^{-\frac{1}{2\sigma_0^2}(\boldsymbol{Y}^*-\boldsymbol{X}\boldsymbol{B})^T(\boldsymbol{Y}^*-\boldsymbol{X}\boldsymbol{B})} d\boldsymbol{B} \\
&= \int_{\boldsymbol{B}} \frac{1}{(2\pi\sigma_0^2)^{\frac{mn}{2}}} e^{-\frac{1}{2\sigma_0^2}\left(\boldsymbol{B}^T\boldsymbol{X}^T\boldsymbol{X}\boldsymbol{B} - 2\boldsymbol{B}^T\boldsymbol{X}^T\boldsymbol{Y}^* + \boldsymbol{Y}^{*T}\boldsymbol{Y}^*\right)} d\boldsymbol{B} \\
&= \int_{\boldsymbol{B}} \frac{1}{(2\pi\sigma_0^2)^{\frac{mn}{2}}} e^{-\frac{1}{2\sigma_0^2}\left\{(\boldsymbol{B}-\widehat{\boldsymbol{B}})^T\boldsymbol{X}^T\boldsymbol{X}(\boldsymbol{B}-\widehat{\boldsymbol{B}}) + \boldsymbol{Y}^{*T}\boldsymbol{Y}^* - \widehat{\boldsymbol{B}}^T\boldsymbol{X}^T\boldsymbol{X}\widehat{\boldsymbol{B}}\right\}} d\boldsymbol{B} \\
&= \frac{1}{(2\pi\sigma_0^2)^{\frac{mn}{2}}} (2\pi\sigma_0^2)^{\frac{mq}{2}} |\boldsymbol{X}^T\boldsymbol{X}|^{-\frac{1}{2}} e^{-\frac{1}{2\sigma_0^2}\left\{\boldsymbol{Y}^{*T}\boldsymbol{Y}^* - \widehat{\boldsymbol{B}}^T\boldsymbol{X}^T\boldsymbol{X}\widehat{\boldsymbol{B}}\right\}} \\
&= |\boldsymbol{X}^T\boldsymbol{X}|^{-\frac{1}{2}} \frac{1}{(2\pi\sigma_0^2)^{\frac{m(n-q)}{2}}} e^{-\frac{1}{2\sigma_0^2}\left\{\boldsymbol{Y}^{*T}\boldsymbol{Y}^* - \widehat{\boldsymbol{B}}^T\boldsymbol{X}^T\boldsymbol{X}\widehat{\boldsymbol{B}}\right\}} \qquad (Here\ \widehat{\boldsymbol{B}} = (\boldsymbol{X}^T\boldsymbol{X})^{-1}\boldsymbol{X}^T\boldsymbol{Y}^*) \\
&= |\boldsymbol{X}^T\boldsymbol{X}|^{-\frac{1}{2}} \frac{1}{(2\pi\sigma_0^2)^{\frac{n-q}{2}}} e^{-\frac{1}{2\sigma_0^2}\left\{\boldsymbol{Y}^{*T}\left(\boldsymbol{I}-\boldsymbol{X}(\boldsymbol{X}^T\boldsymbol{X})^{-1}\boldsymbol{X}^T\right)\boldsymbol{Y}^*\right\}}
\end{aligned} \tag{1}
$$

The marginal distribution for $\boldsymbol{Y}^*$ can be simplified as:

$$
p(\boldsymbol{Y}^* \mid \sigma_0^2) \propto (\sigma_0^2)^{-\frac{m(n-q)}{2}} \exp\left(tr\left(-\frac{\boldsymbol{Y}^{*T}\boldsymbol{M}\boldsymbol{Y}^*}{2\sigma_0^2}\right)\right). \tag{2}
$$

The distribution for $\boldsymbol{Y}$, conditional on $\boldsymbol{Z}$, thus becomes:

$$
p(\boldsymbol{Y} \mid \boldsymbol{Z}, \boldsymbol{W}, \sigma_0^2, \tau) \propto (\sigma_0^2)^{-\frac{m(n-q)}{2}} \exp\left(tr\left(-\frac{(\boldsymbol{Y} - \boldsymbol{W}\boldsymbol{Z})\boldsymbol{M}(\boldsymbol{Y} - \boldsymbol{W}\boldsymbol{Z})^T}{2\sigma_0^2}\right)\right). \tag{3}
$$

The joint likelihood of $\boldsymbol{Y}, \boldsymbol{Z}$ is

$$
\begin{aligned}
&p(\boldsymbol{Y}, \boldsymbol{Z} \mid \boldsymbol{W}, \sigma_0^2, \tau) \propto p(\boldsymbol{Y} \mid \boldsymbol{Z}, \boldsymbol{W}, \sigma_0^2, \tau) p(\boldsymbol{Z} | \boldsymbol{W}, \sigma_0^2, \tau) \\
&\propto (\sigma_0^2)^{-\frac{m(n-q)}{2}} \prod_{l=1}^{d} |\sigma_0^2 \tau \boldsymbol{K}|^{-\frac{1}{2}} \exp\left(tr\left(-\frac{(\boldsymbol{Y} - \boldsymbol{W}\boldsymbol{Z})\boldsymbol{M}(\boldsymbol{Y} - \boldsymbol{W}\boldsymbol{Z})^T + \sum_{l=1}^{d} \boldsymbol{Z}_l (\tau\boldsymbol{K})^{-1} \boldsymbol{Z}_l^T}{2\sigma_0^2}\right)\right) \\
&\propto (\sigma_0^2)^{-\frac{m(n-q)}{2}} \prod_{l=1}^{d} |\sigma_0^2 \tau \boldsymbol{K}|^{-\frac{1}{2}} \exp\left(tr\left(-\frac{\boldsymbol{Y}\boldsymbol{M}\boldsymbol{Y}^T}{2\sigma_0^2}\right)\right) \\
&\times \exp\left\{-\frac{\boldsymbol{Z}\boldsymbol{M}\boldsymbol{Z}^T - 2\boldsymbol{Z}^T(\boldsymbol{W}^T\boldsymbol{M})\boldsymbol{Y} + \sum_{l=1}^{d} \boldsymbol{Z}_l (\tau\boldsymbol{K})^{-1} \boldsymbol{Z}_l^T}{2\sigma_0^2}\right\}.
\end{aligned}
$$

$$(4)$$

Next, we integrate out $\boldsymbol{Z}$ to obtain the marginal distribution for $\boldsymbol{Y}$:

$$\begin{aligned}
&p(\boldsymbol{Y}|\boldsymbol{W},\sigma_0^2,\tau) \\
&\propto (\sigma_0^2)^{-\frac{m(n-q)}{2}} \prod_{l=1}^{d} |\tau\boldsymbol{MK}+\boldsymbol{I}_n|^{-\frac{1}{2}} \exp\left(tr\left(-\frac{\boldsymbol{YMY}^T}{2\sigma_0^2}\right)\right) \\
&\times \exp\left\{-\frac{\sum_{l=1}^{d}\boldsymbol{Y}^T(\boldsymbol{W}^T\boldsymbol{M})^T(\boldsymbol{M}+\tau^{-1}\boldsymbol{K}^{-1})^{-1}(\boldsymbol{W}^T\boldsymbol{M})\boldsymbol{Y}}{2\sigma_0^2}\right\} \\
&\propto (\sigma_0^2)^{-\frac{m(n-q)}{2}} \prod_{l=1}^{d} |\tau\boldsymbol{MK}+\boldsymbol{I}_n|^{-\frac{1}{2}} \\
&\times \exp\left\{-\frac{\mathrm{tr}(\boldsymbol{YMY}^T)-\sum_{l=1}^{d}\boldsymbol{w}_l^T\boldsymbol{YM}(\boldsymbol{M}+\tau^{-1}\boldsymbol{K}^{-1})^{-1}\boldsymbol{MY}^T\boldsymbol{w}_l}{2\sigma_0^2}\right\}.
\end{aligned} \tag{5}$$

Based on the marginal distribution of $\boldsymbol{Y}$, we can obtain the maximum likelihood estimator of σ_0^2 as

$$\hat{\sigma}_0^2 = \frac{tr\,(\boldsymbol{YMY}^T)-\sum_{l=1}^{d}\boldsymbol{w}_l^T\boldsymbol{YM}(\boldsymbol{M}+\tau^{-1}\boldsymbol{K}^{-1})^{-1}\boldsymbol{MY}^T\boldsymbol{w}_l}{m(n-q)}. \tag{6}$$

Above, $\boldsymbol{w}_l$ is the l-th column of the loading matrix $\boldsymbol{W}$.

Denote $S = tr\,(\boldsymbol{YMY}^T)-\sum_{l=1}^{d}\boldsymbol{w}_l^T\boldsymbol{YM}(\boldsymbol{M}+\tau^{-1}\boldsymbol{K}^{-1})^{-1}\boldsymbol{MY}^T\boldsymbol{w}_l$, plugging the maximum likelihood estimator of σ_0^2 back to the marginal distribution of $\boldsymbol{Y}$ in equation (5) gives

$$\begin{aligned}
&p(\boldsymbol{Y}|\boldsymbol{W},\hat{\sigma}_0^2,\tau) \\
&\propto |S|^{-\frac{m(n-q)}{2}} \prod_{l=1}^{d}\{|\tau\boldsymbol{MK}+\boldsymbol{I}_n|\}^{-\frac{1}{2}} \\
&\propto |S|^{-\frac{m(n-q)}{2}} \prod_{l=1}^{d}\{|\tau\boldsymbol{K}+\boldsymbol{I}_n||\boldsymbol{I}_n-(\tau\boldsymbol{K}+\boldsymbol{I}_n)^{-1}\tau\boldsymbol{KX}(\boldsymbol{X}^T\boldsymbol{X})^{-1}\boldsymbol{X}^T|\}^{-\frac{1}{2}} \\
&\propto |S|^{-\frac{m(n-q)}{2}} \prod_{l=1}^{d}\{|\tau\boldsymbol{K}+\boldsymbol{I}_n||\boldsymbol{X}^T\boldsymbol{X}|^{-1}|\boldsymbol{X}^T(\tau\boldsymbol{K}+\boldsymbol{I}_n)^{-1}\boldsymbol{X}|\}^{-\frac{1}{2}} \\
&\propto |S|^{-\frac{m(n-q)}{2}} \prod_{l=1}^{d}\{|\tau\boldsymbol{K}+\boldsymbol{I}_n|^{-\frac{1}{2}}|\boldsymbol{X}^T(\tau\boldsymbol{K}+\boldsymbol{I}_n)^{-1}\boldsymbol{X}|^{-\frac{1}{2}}\}.
\end{aligned} \tag{7}$$

We further maximize the above marginal distribution to obtain the estimators for $\boldsymbol{W}$ and τ:

$$\widehat{\boldsymbol{W}} = \underset{\boldsymbol{W}}{argmax} \sum_{l=1}^{d}\boldsymbol{w}_l^T\boldsymbol{YM}(\boldsymbol{M}+\tau^{-1}\boldsymbol{K}^{-1})^{-1}\boldsymbol{MY}^T\boldsymbol{w}_l,\ \ s.t.\ \ \boldsymbol{W}^T\boldsymbol{W}=\boldsymbol{I}_d, \tag{8}$$

$$\hat{\tau} = \underset{\tau}{argmax}\, p(\boldsymbol{Y} \mid \widehat{\boldsymbol{W}},\tau). \tag{9}$$

We use the Brent's optimization method implemented in the *optim* function in R to obtain the estimation of τ in equation (9). We obtain the closed-form expression[1,2] of $\widehat{\boldsymbol{W}}$ in the form of $\widehat{\boldsymbol{W}}=\boldsymbol{LR}$, where $\boldsymbol{L}$ is a m by d matrix for the first d eigenvectors of $\boldsymbol{YM}(\boldsymbol{M}+\tau^{-1}\boldsymbol{K}^{-1})^{-1}\boldsymbol{MY}^T$and $\boldsymbol{R}$ is an arbitrary d by d orthogonal rotation matrix.

In addition, based on equation (4), we can obtain the maximum likelihood estimate for each $\boldsymbol{Z}_l$ as

$$\boldsymbol{Z}_l|\boldsymbol{Y},\widehat{\boldsymbol{W}},\hat{\sigma}_0^2,\hat{\tau} \sim MVN\left(\widehat{\boldsymbol{Z}}_l,\widehat{\boldsymbol{\Sigma}}_{Z_l}\right), \tag{10}$$

where $\widehat{\boldsymbol{Z}}_l = (\boldsymbol{M} + \hat{\tau}^{-1}\boldsymbol{K}^{-1})^{-1}\boldsymbol{M}\boldsymbol{Y}^T\widehat{\boldsymbol{w}_l}$, and $\widehat{\boldsymbol{\Sigma}}_{Z_l} = \hat{\sigma}_0^2(\boldsymbol{M} + \hat{\tau}^{-1}\boldsymbol{K}^{-1})^{-1}$. From equation (1) we can further obtain $\widehat{\boldsymbol{B}} = (\boldsymbol{X}^T\boldsymbol{X})^{-1}\boldsymbol{X}^T(\boldsymbol{Y}^T - \widehat{\boldsymbol{Z}}^T\widehat{\boldsymbol{W}}^T)$.

Carrying out the above inference algorithm requires calculating the following three quantities: $(\boldsymbol{M} + \tau^{-1}\boldsymbol{K}^{-1})^{-1}$ in equations (6), (8) and (10), as well as $|\tau\boldsymbol{K} + \boldsymbol{I}_n|$ and $|\boldsymbol{X}^T(\tau\boldsymbol{K} + \boldsymbol{I}_n)^{-1}\boldsymbol{X}|$ in equation (7). Each quantity in equations (6)-(8) needs to be re-evaluated in each iteration of the inference algorithm as τ is being updated while the quantity in equation (10) needs to be evaluated once at the last iteration. To improve the computation efficiency of the above inference algorithm, we first perform eigen decomposition on the kernel matrix $\boldsymbol{K} = \boldsymbol{U}\boldsymbol{D}\boldsymbol{U}^T$ at the beginning of the optimization, where $\mathbf{D} = diag(\delta_1, \dots, \delta_n)$ with δ_i being the eigen values, and $\boldsymbol{U}$ is the eigenvector matrix. With the eigen decomposition of $\boldsymbol{K}$, we can simplify the calculation of the three quantities.

Specifically, for $(\boldsymbol{M} + \tau^{-1}\boldsymbol{K}^{-1})^{-1}$, it can be calculated using the Woodbury formula as:

$$\begin{aligned}
&(\boldsymbol{M} + \tau^{-1}\boldsymbol{K}^{-1})^{-1} \\
&= (\boldsymbol{I} - \boldsymbol{X}(\boldsymbol{X}^T\boldsymbol{X})^{-1}\boldsymbol{X}^T + \tau^{-1}\boldsymbol{K}^{-1})^{-1} \\
&= (\boldsymbol{I} + \tau^{-1}\boldsymbol{K}^{-1} + \boldsymbol{X}(-(\boldsymbol{X}^T\boldsymbol{X})^{-1})\boldsymbol{X}^T)^{-1} \\
&= (\boldsymbol{I} + \tau^{-1}\boldsymbol{K}^{-1})^{-1} - (\boldsymbol{I} + \tau^{-1}\boldsymbol{K}^{-1})^{-1}\boldsymbol{X}[-\boldsymbol{X}^T\boldsymbol{X} + \boldsymbol{X}^T(\boldsymbol{I} + \tau^{-1}\boldsymbol{K}^{-1})^{-1}\boldsymbol{X}]^{-1}\boldsymbol{X}^T(\boldsymbol{I} + \tau^{-1}\boldsymbol{K}^{-1})^{-1}.
\end{aligned}$$

where

$$\begin{aligned}
&(\boldsymbol{I} + \tau^{-1}\boldsymbol{K}^{-1})^{-1} \\
&= (\boldsymbol{I} + \boldsymbol{U}(\tau^{-1}\boldsymbol{D}^{-1})\boldsymbol{U}^T)^{-1} \\
&= \boldsymbol{U}(\boldsymbol{I} + \tau^{-1}\boldsymbol{D}^{-1})^{-1}\boldsymbol{U}^T
\end{aligned} \tag{11}$$

Therefore, we can compute $\boldsymbol{U}^T\boldsymbol{M}\boldsymbol{Y}^T$ at the beginning of the algorithm and then evaluate the quantities in equations (6) and (8) in each iteration of algorithm with a linear complexity with respect to *n*. In addition, we can evaluate $(\boldsymbol{M} + \tau^{-1}\boldsymbol{K}^{-1})^{-1}\boldsymbol{M}\boldsymbol{Y}^T$ in equation (10) in the last iteration with a quadratic complexity with respect to *n*.

For $|\tau\boldsymbol{K} + \boldsymbol{I}_n|$, we can express it as $|\tau\boldsymbol{K} + \boldsymbol{I}_n| = |\tau\boldsymbol{D} + \boldsymbol{I}_n|$, which has a linear complexity with respect to *n*. For $|\boldsymbol{X}^T(\tau\boldsymbol{K} + \boldsymbol{I}_n)^{-1}\boldsymbol{X}|$, we first calculate $(\tau\boldsymbol{K} + \boldsymbol{I}_n)^{-1} = \boldsymbol{U}(\boldsymbol{I}_n + \tau\boldsymbol{D})^{-1}\boldsymbol{U}^T$ and then calculate $(\tau\boldsymbol{K} + \boldsymbol{I}_n)^{-1}\boldsymbol{X}$ and $\boldsymbol{X}^T(\tau\boldsymbol{K} + \boldsymbol{I}_n)^{-1}\boldsymbol{X}$ before taking its determinant. Because we can compute $\boldsymbol{U}^T\boldsymbol{X}$ at the beginning of the algorithm, evaluating $|\boldsymbol{X}^T(\tau\boldsymbol{K} + \boldsymbol{I}_n)^{-1}\boldsymbol{X}|$ in each iteration has a linear complexity with respect to *n*.

To further facilitate computation, we applied low-rank approximation in the eigen decomposition step for $\boldsymbol{K}$. In particular, we use the "RSpectra" R package in the eigen decomposition to only obtain the top *r* eigenvectors and *r* eigenvalues. Therefore, both $\boldsymbol{U}$ and $\boldsymbol{D}$ become low-rank matrices with dimension *n* by *r* for $\boldsymbol{U}$ and *r* by *r* for $\boldsymbol{D}$. In this case, we calculate $(\boldsymbol{I} + \tau^{-1}\boldsymbol{K}^{-1})^{-1}$ in equation (11) as

$$(\boldsymbol{I} + \tau^{-1}\boldsymbol{K}^{-1})^{-1} = \boldsymbol{I} - \boldsymbol{U}(\tau\boldsymbol{D} + \boldsymbol{U}^T\boldsymbol{U})^{-1}\boldsymbol{U}^T, \tag{12}$$

and evaluate $\widehat{\boldsymbol{Z}}$ in equation (10) as

$$\widehat{\boldsymbol{Z}} = \widehat{\boldsymbol{W}}^T\boldsymbol{Y}\boldsymbol{M}(\boldsymbol{M} + \tau^{-1}\boldsymbol{K}^{-1})^{-1} = \hat{\tau}\widehat{\boldsymbol{W}}^T\boldsymbol{Y}\boldsymbol{M}\boldsymbol{K} - \hat{\tau}\widehat{\boldsymbol{W}}^T\boldsymbol{Y}\boldsymbol{M}\boldsymbol{U}(\tau^{-1}\boldsymbol{D}^{-1} + \boldsymbol{U}^T\boldsymbol{M}\boldsymbol{U})^{-1}\boldsymbol{U}^T\boldsymbol{M}\boldsymbol{K}. \tag{13}$$

In the above low-rank approximation, we choose the rank *r* as a function of sample size. Specifically, for data with a large sample size (n>5,000), we evaluated quantities in equations (6) and (8) in each iteration using a small *r*=20, since these quantities are insensitive to the choice of rank *r*. We obtained the estimates $\widehat{\boldsymbol{Z}}_l$ in equation (10) using a relatively large *r*, with *r* set to be 10% of the sample size *n*, which ensures the top *r* eigen values to explain at least 90% of the variance in the present study. For data with a small sample size (n≤5,000), we use the same *r*

to evaluate quantities in equations (6), (8) and (10), with r chosen to ensure that the top r eigen values explain at least 90% of the variance. Our software implementation also allows users to specify their own choice of r.

Overall, the computational time complexity of our algorithm is $O(tdm^2+rn^2)$, where t represents the number of iterations in the optimization algorithm, with memory requirement being $O(mn+n^2)$.

Supplementary Figures

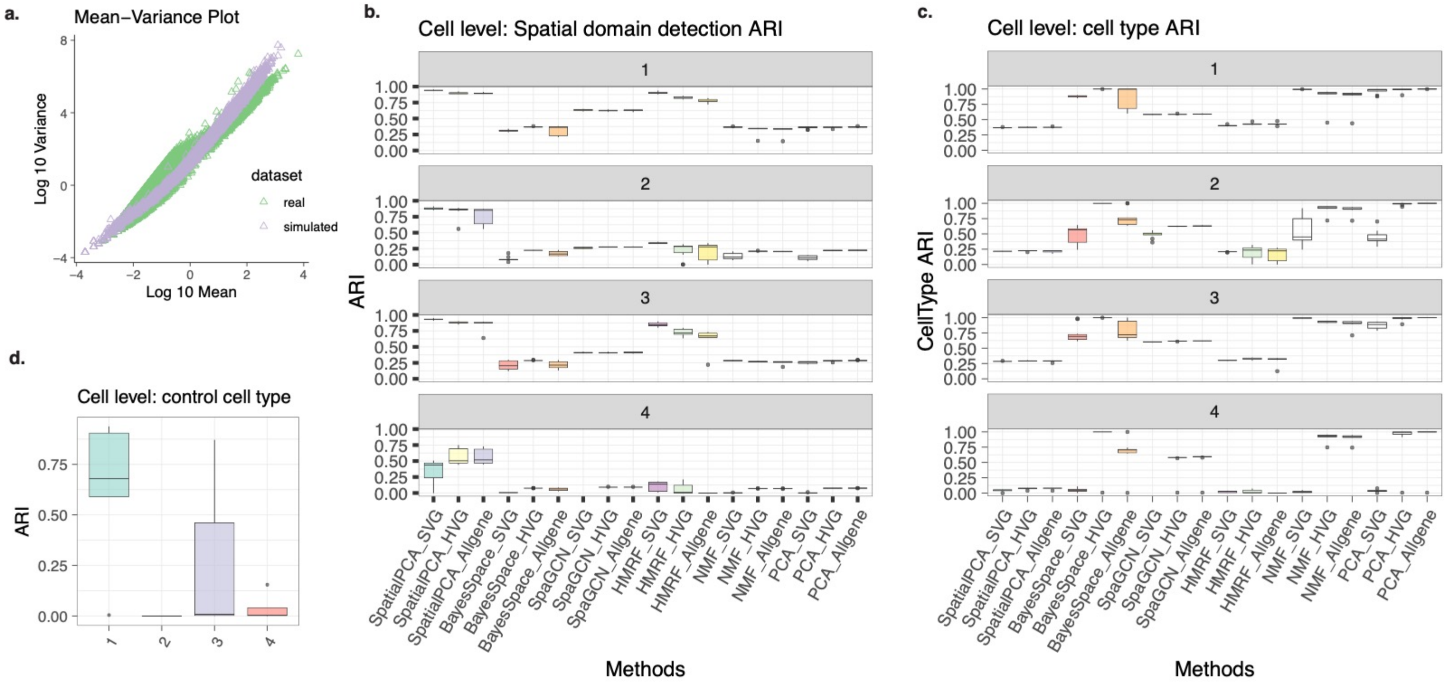

Supplementary Figure 1. Simulation results for spatial domain clustering and cell type clustering in single cell resolution. a. The mean and variance relationship between real scRNA-seq count data and simulated single cell count data are consistent. **b**. Spatial domain clustering results using different methods paired with spatially variable genes (SVGs), highly variable genes (HVGs) and all genes in four simulation scenarios (n=10,000 cells). **c**. Cell type clustering results using different methods (n=10,000 cells). In SpatialPCA, we aim to identify spatial domains. In the simulations, SpatialPCA has highest adjusted Rand index (ARI, the higher the better) in spatial domain clustering and lowest ARI in cell type clustering, highlights the different goals in spatial domain and cell type detection. **d**. After controlling for cell types as covariates in SpatialPCA, the clustering performance is the best when there is one dominant cell type in each spatial domain (scenario 1), and the performance reduces when there are multiple cell types mixed in each spatial domain (scenario 2, 3 and 4, sample size n=10,000 cells). In the boxplots in **b-d**, the center line, box limits and whiskers denote the median, upper and lower quartiles, and 1.5× interquartile range, respectively. These results highlight the spatial domains are driven by the cell type compositions.

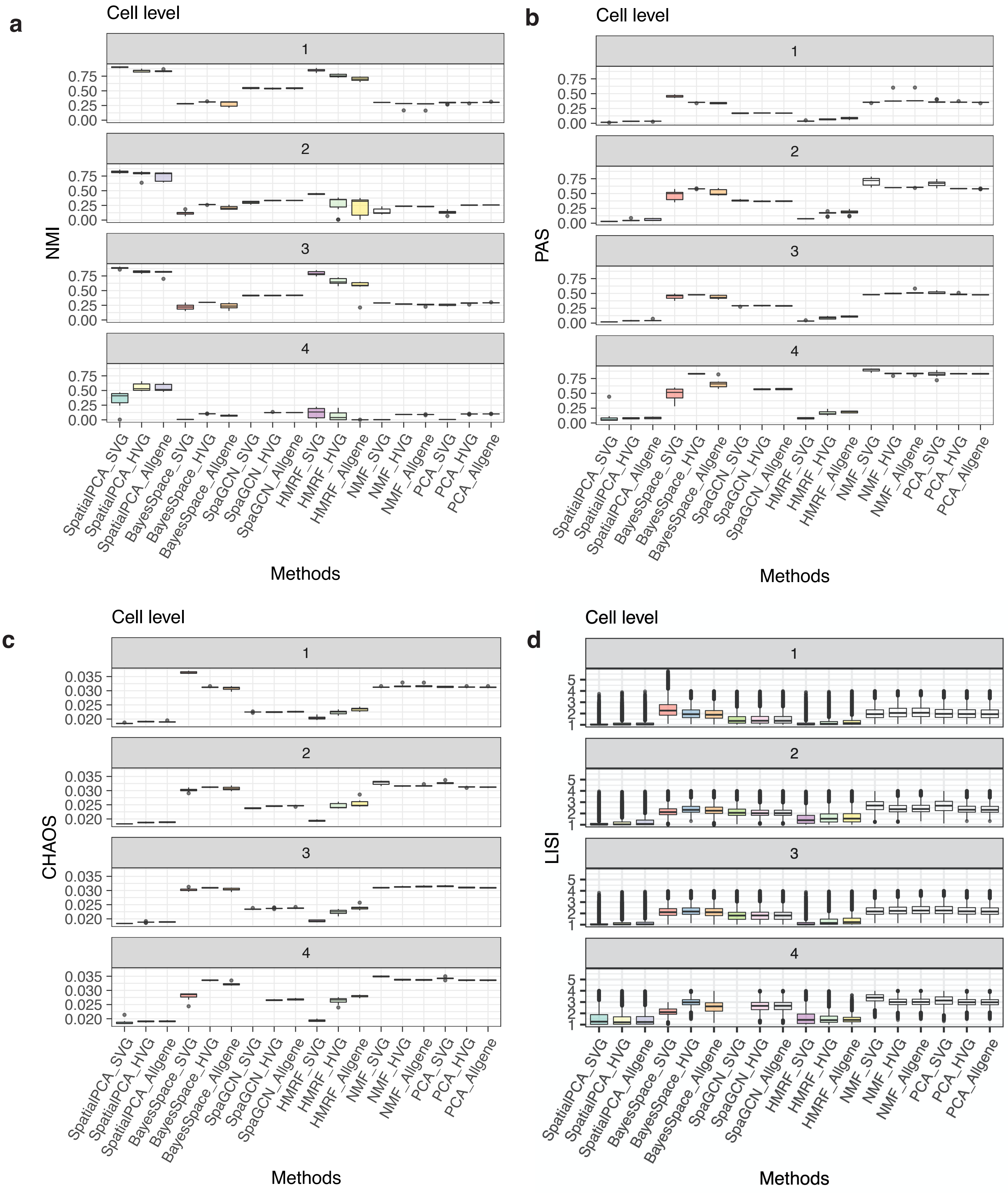

Supplementary Figure 2. Simulation results for spatial domain clustering in single cell resolution. Spatial domain clustering results using different methods paired with SVGs, HVGs and all genes in four simulation scenarios (n=10,000 cells), in terms of **a**. Normalized mutual information (NMI, the higher the better), **b**.

Percentage of abnormal spots (PAS, the lower the better), **c**. Spatial chaos score (CHAOS, the lower the better), and **d**. Local inverse Simpson's index (LISI, the lower the better) scores. In the boxplots in **a-d**, the center line, box limits and whiskers denote the median, upper and lower quartiles, and 1.5× interquartile range, respectively.

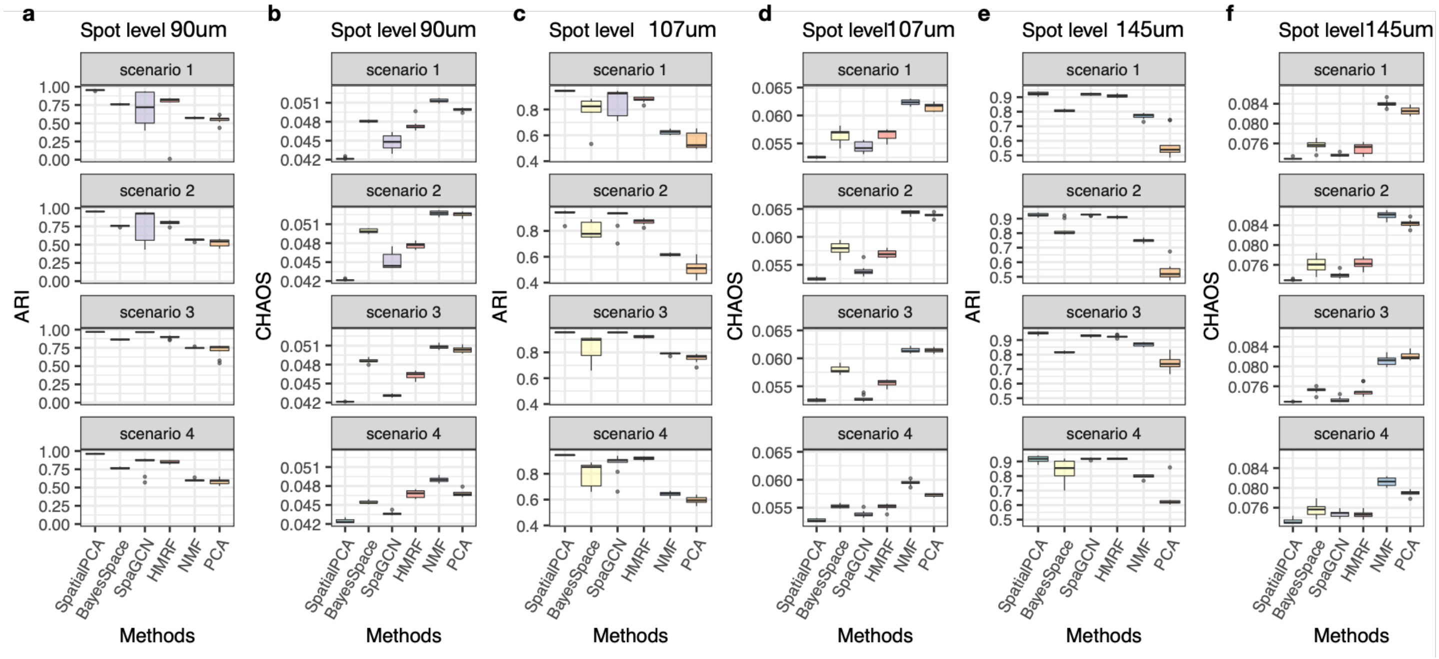

Supplementary Figure 3. Simulation results for spatial domain clustering at spot level. a-b. Spatial domain clustering results in adjusted Rand index (ARI, the higher the better) and spatial chaos score (CHAOS, the lower the better) using different methods at spot diameter being 90um (n=5,077 spots). **c-d**. Spatial domain clustering results in ARI and CHAOS using different methods at spot diameter being 107um (n=3,602 spots). **e-f**. Spatial domain clustering results in ARI and CHAOS using different methods at spot diameter being 145um (n=1,948 spots). In the boxplots in **a**-**f**, the center line, box limits and whiskers denote the median, upper and lower quartiles, and 1.5× interquartile range, respectively.

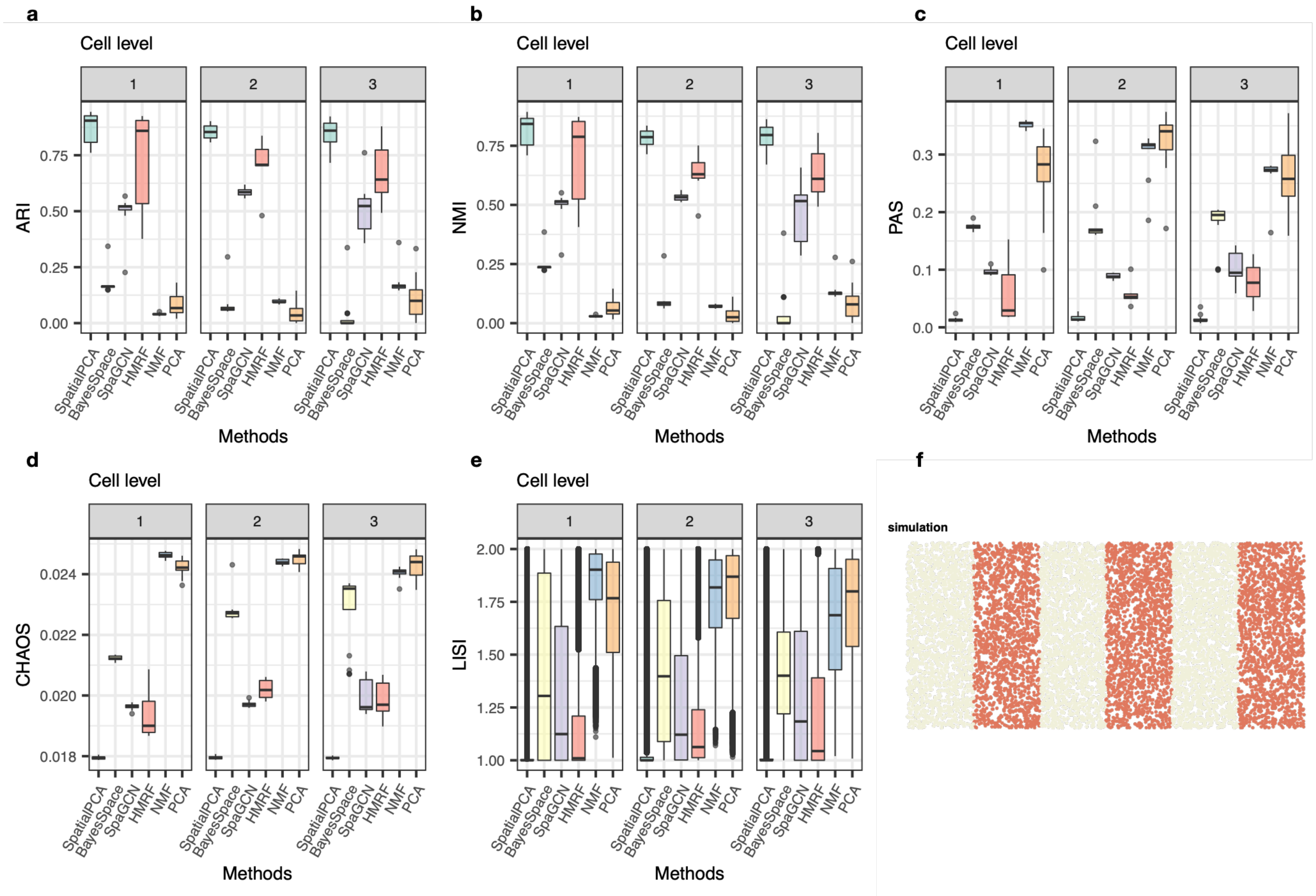

Supplementary Figure 4. Simulation results for spatial domain clustering at cell level with stripe pattern. Spatial domain clustering results using different methods in terms of **a**. Adjusted Rand index (ARI), left: scenario 1; middle: scenario 2; right: scenario 3. **b**. Normalized mutual information (NMI, the higher the better), **c**. Percentage of abnormal spots (PAS, the lower the better), **d**. Spatial chaos score (CHAOS, the lower the better) and **e**. Local inverse Simpson's index (LISI, the lower the better) in 10,000 cells. **f**. The designed ground truth stripe pattern. In the boxplots in **a**-**e**, the center line, box limits and whiskers denote the median, upper and lower quartiles, and 1.5× interquartile range, respectively.

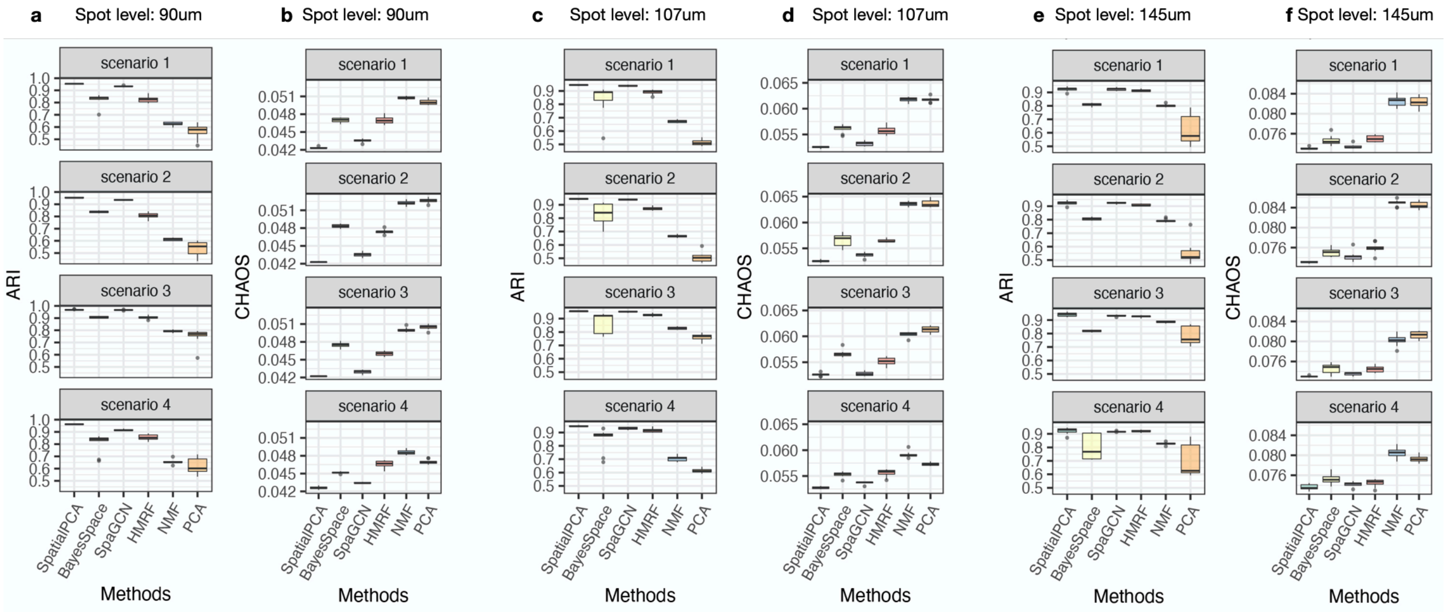

Supplementary Figure 5. Simulation results for spatial domain clustering at spot level with artifactual spatial correlation between spots. a-b. Spatial domain clustering results using different methods at spot diameter being 90um (n=5,077 spots) in terms of adjusted Rand index (ARI, the higher the better) and spatial chaos score (CHAOS, the lower the better). **c-d**. Spatial domain clustering results using different methods at spot diameter being 107um (n=3,602 spots) in terms of ARI and CHAOS. **e-f**. Spatial domain clustering results using different methods at spot diameter being 145um (n=1,948 spots) in terms of ARI and CHAOS. In the boxplots in **a-f**, the center line, box limits and whiskers denote the median, upper and lower quartiles, and 1.5× interquartile range, respectively.

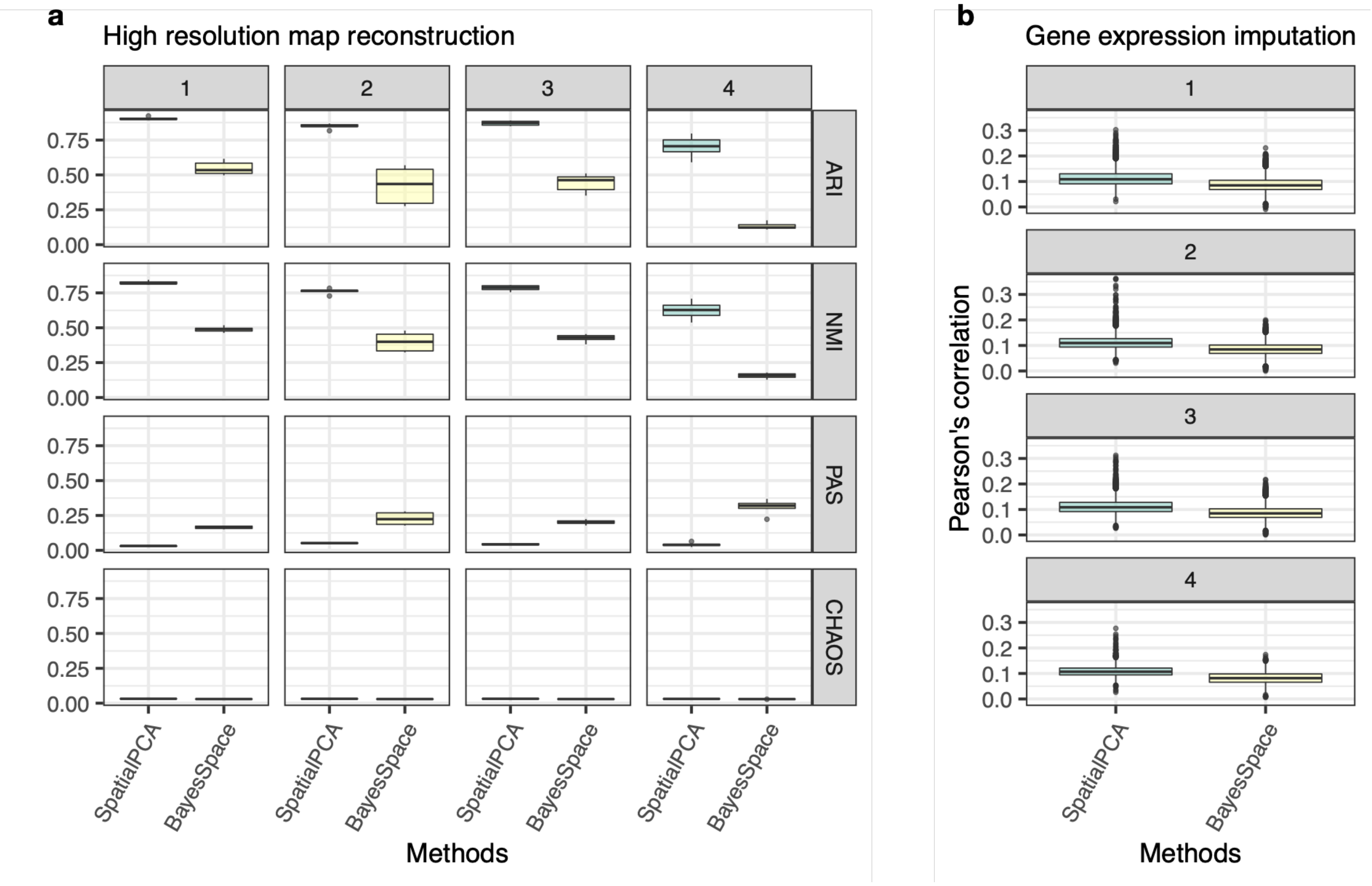

Supplementary Figure 6. High resolution spatial map reconstruction and gene expression imputation simulation. a. High resolution spatial map clustering results for spot level simulation in four scenarios at spot diameter being 145um (n=1,948 spots). We compared SpatialPCA with BayesSpace in terms of adjusted Rand index (ARI, the higher the better), normalized mutual information (NMI, the higher the better), percentage of abnormal spots (PAS, the lower the better) and spatial chaos score (CHAOS, the lower the better). **b**. High resolution gene expression prediction results for spot level simulation in four scenarios at spot diameter being 145um (n=1,948 spots). We compared SpatialPCA with BayesSpace for the Pearson's correlation between predicted gene expression with ground truth expression. In the boxplots in **a-b**, the center line, box limits and whiskers denote the median, upper and lower quartiles, and 1.5× interquartile range, respectively.

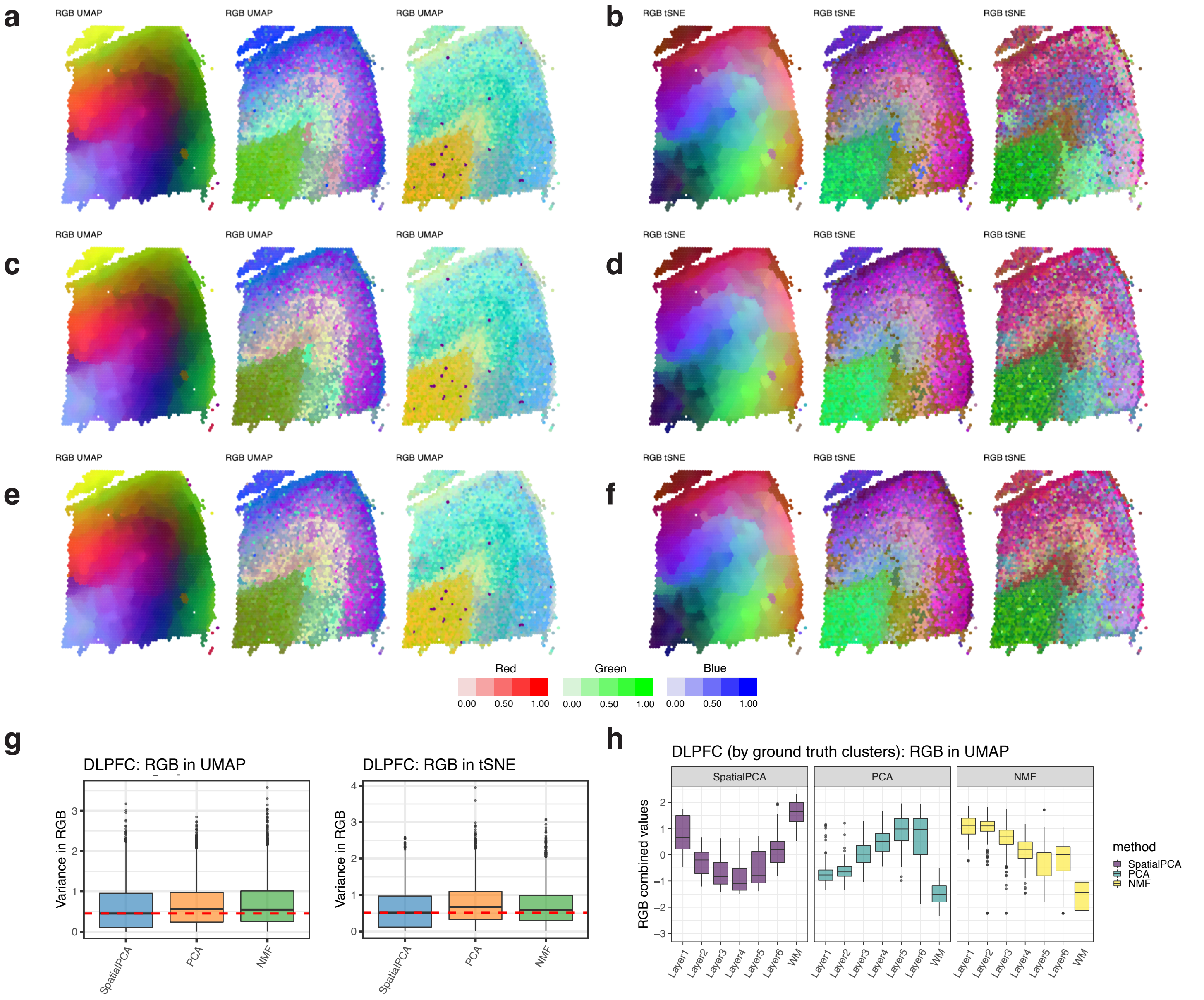

Supplementary Figure 7. RGB plots for the DLPFC data. **a-f**. For SpatialPCA, PCA, and NMF, we summarized the inferred low dimensional components into three UMAP (**a, c, e**) or tSNE components (**b, d, f**) and visualized the three resulting components with red/green/blue (RGB) colors through the RGB plot. Color code corresponds to the RGB values of each location's three UMAP or tSNE components inferred from low dimensional components in dimension reduction. Different colors indicate different values for each of the three UMAP or tSNE components on the tissue section, highlighting the difference of the low dimensional components from different methods included in the panel. The RGB plot from SpatialPCA displays laminar organization of the cortex and show less color differences within a local area. We also scaled up spatial PCs/regular PCs 10 times (**c-d**) and 20 times (**e-f**) to see the influence of range of the PCs to RGB plots. The tSNE/UMAP results and RGB plots in figures (**c-f**) have very similar patterns as shown at the original scale (**a-b**). **g**. We found in SpatialPCA, the weighted RGB values have lower variance than PCA or NMF in nearby spots (n=3,460 spots). **h.** The RGB plots in SpatialPCA show a smoother transition of colors between adjacent cortical layers (n=3,460 spots). The cortical layers are labeled based on ground truth annotations. In the boxplots in **g-h**, the center line, box limits and whiskers denote the median, upper and lower quartiles, and 1.5× interquartile range, respectively.

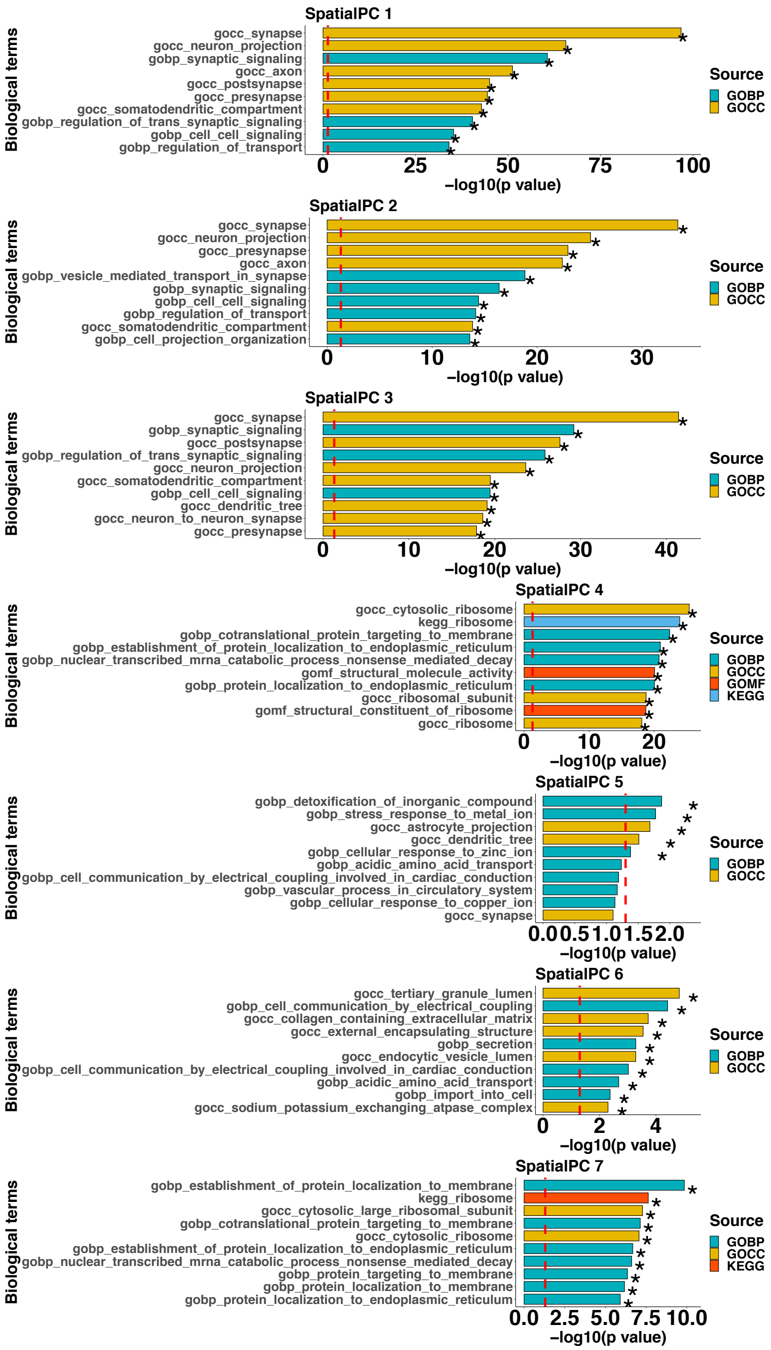

Supplementary Figure 8. Gene set enrichment analysis on the genes associated with Spatial PC values in the DLPFC data sample 151676. The top 10 enriched gene sets are shown for each of the top 7 spatial PCs. No significant genes were detected in the rest 13 spatial PCs. Color represents different data sources for annotating the gene sets. The enrichment is given as -log10 adjusted p-value (g:SCS correction, details in Methods) of the genes associated with spatial PC values.

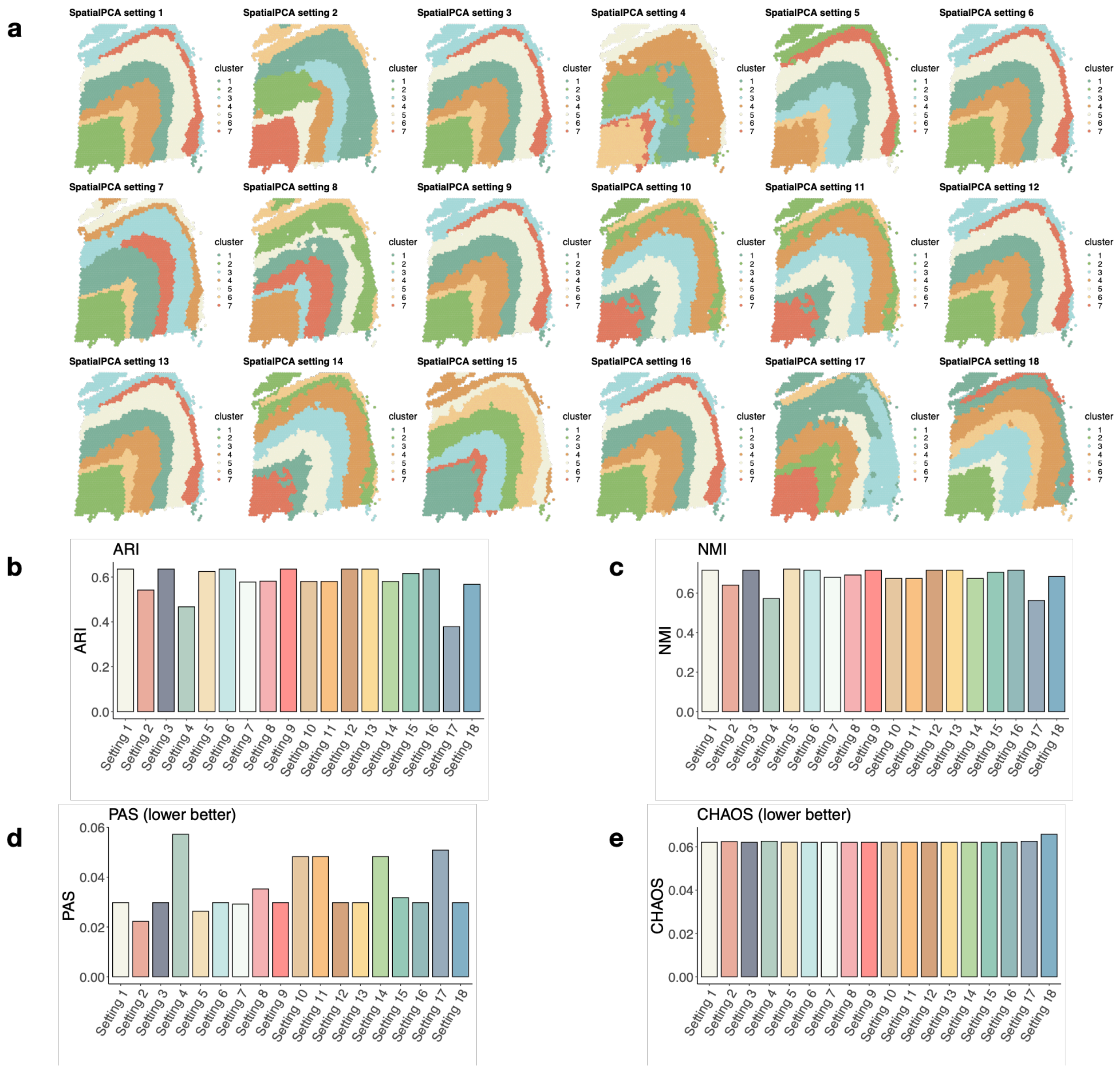

Supplementary Figure 9. Sensitivity analyses for the DLPFC data. **a**. Clustering results from SpatialPCA are shown for different analytic settings. Setting 1: using top 3000 spatially variable genes detected by SPARK. Setting 2: using top 1000 spatially variable genes detected by SPARK. Setting 3: using all spatially variable genes detected by SPARK. Setting 4: using all highly variable genes detected by Seurat. Setting 5: using top 10 Spatial PCs. Setting 6: using top 20 Spatial PCs. Setting 7: using top 30 Spatial PCs. Setting 8: using top 50 Spatial PCs. Setting 9: using Gaussian kernel. Setting 10: using Cauchy kernel. Setting 11: using quadratic kernel. Setting 12: controlling for cell types when selecting SVGs in SPARK-X. Setting 13: controlling for cell types in SpatialPCA. Setting 14: controlling for cell types by regressing them out from the input gene expression and taking the residuals. Setting 15: controlling for cell density in the spots. Setting 16: gene expression normalized through SCTransform normalization. Setting 17: gene expression normalized through log normalization. Setting 18: taking the histology information as a third dimension in location matrix. **b**. Clustering accuracy as measured by adjusted Rand index (ARI, the higher the better) for different settings. **c**. Clustering accuracy as measured by normalized mutual information (NMI, the higher the better) for different settings. **d**. Spatial continuity of the inferred clusters as measured by percentage of abnormal spots (PAS, the lower the better) for different settings. **e**. Spatial continuity of the inferred clusters as measured by spatial chaos score (CHAOS, the lower the better) for different settings.

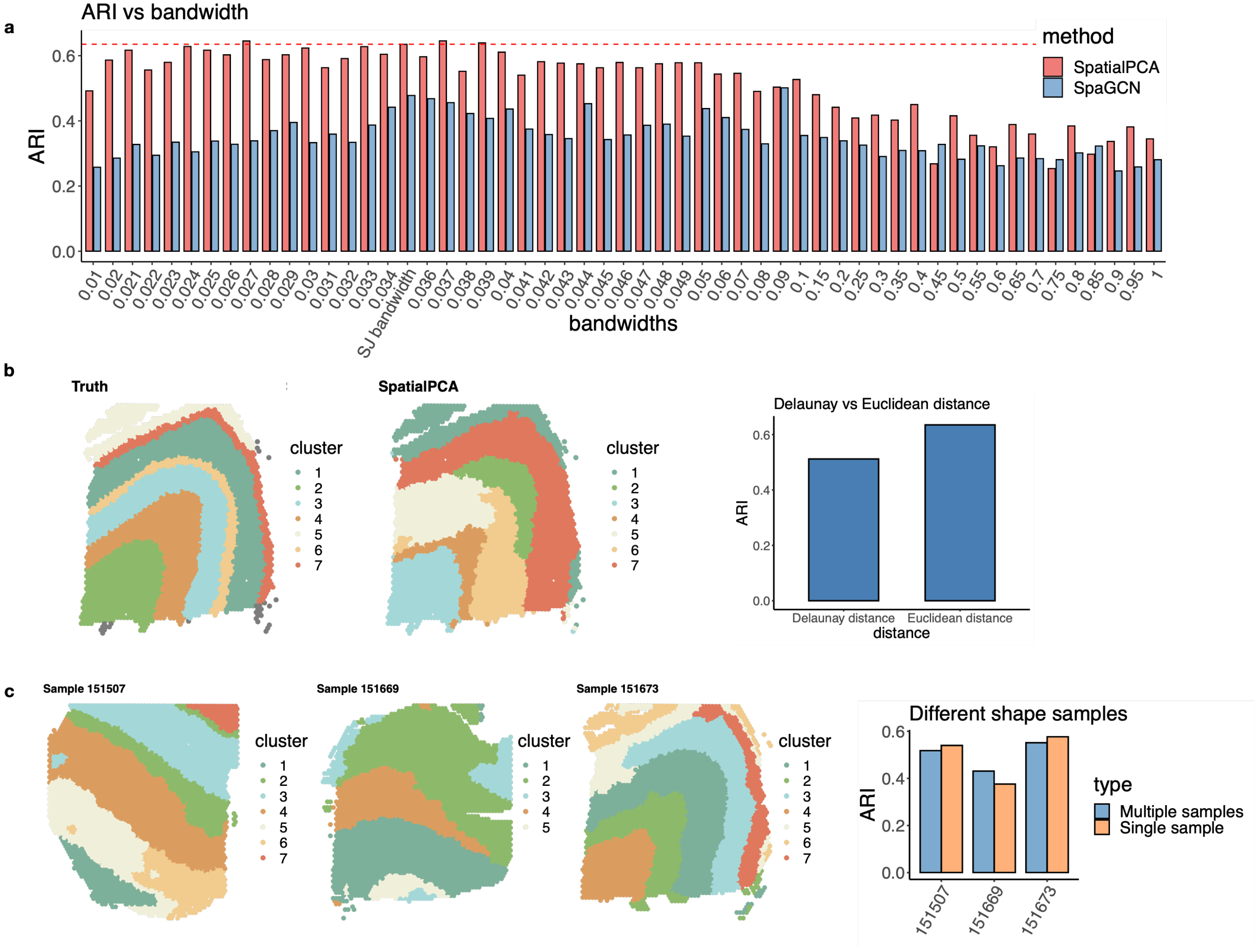

Supplementary Figure 10. Additional sensitivity analyses for the DLPFC data. **a**. The spatial clustering results from SpatialPCA and SpaGCN as measured by adjusted Rand index (ARI, the higher the better, y-axis) across different values of the bandwidth parameter (x-axis) in sample 151676. Results are obtained using the Gaussian kernel, with the red dash line representing the ARI value at the default bandwidth obtained from the SJ method. **b.** Left: visualization of the spatial domains detected in SpatialPCA with a distance matrix calculated from Delaunay triangulation. Right: comparison of spatial clustering accuracy measured by ARI for using Delaunay distance versus using the Euclidian distance. **c.** Left: spatial clusters obtained by jointly modeling multiple tissue sections (samples 151507, 151069, and 151673) using SpatialPCA. Right: comparison of spatial clustering accuracy by ARI for joint modeling of multiple tissue sections versus modeling each section separately.

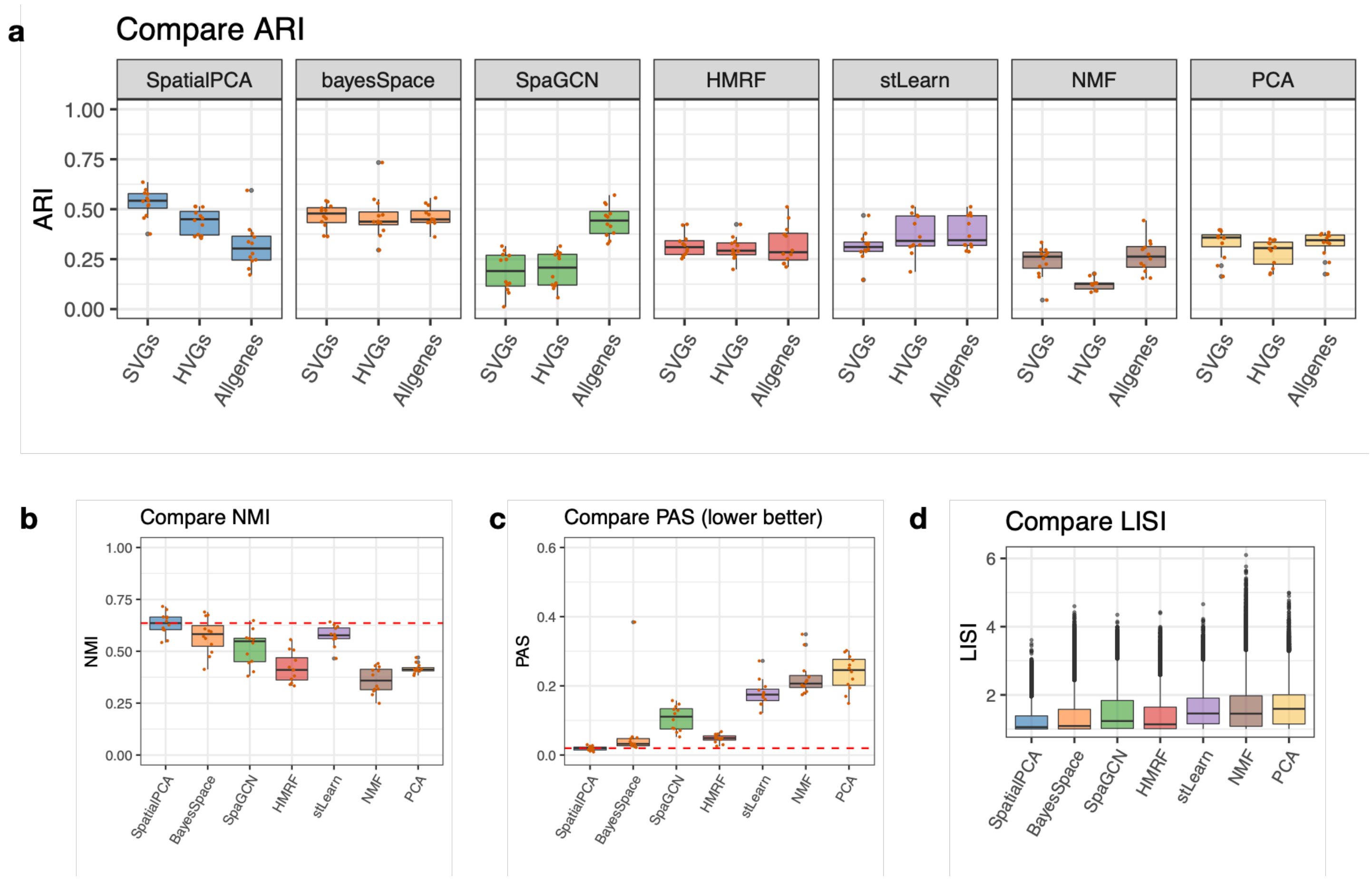

Supplementary Figure 11. Clustering results obtained based on different methods in the DLPFC data. a. Clustering results measured by adjusted Rand index (ARI, the higher the better) in all 12 sections. In dimension reduction methods (SpatialPCA, PCA, and NMF), clustering was performed based on the inferred low-dimensional components. For spatial domain clustering methods (BayesSpace, SpaGCN and HMRF), clustering was performed based the default settings. All the methods are paired with SVGs, HVGs and all genes. **b-d**. Clustering results measured by normalized mutual information (NMI, the higher the better), percentage of abnormal spots (PAS, the lower the better), and local inverse Simpson's index (LISI, the lower the better) in different methods with their default settings in all 12 sections. Clustering results of PCA and NMF are obtained with SVGs. In the boxplots in **a-d**, the center line, box limits and whiskers denote the median, upper and lower quartiles, and 1.5× interquartile range, respectively.

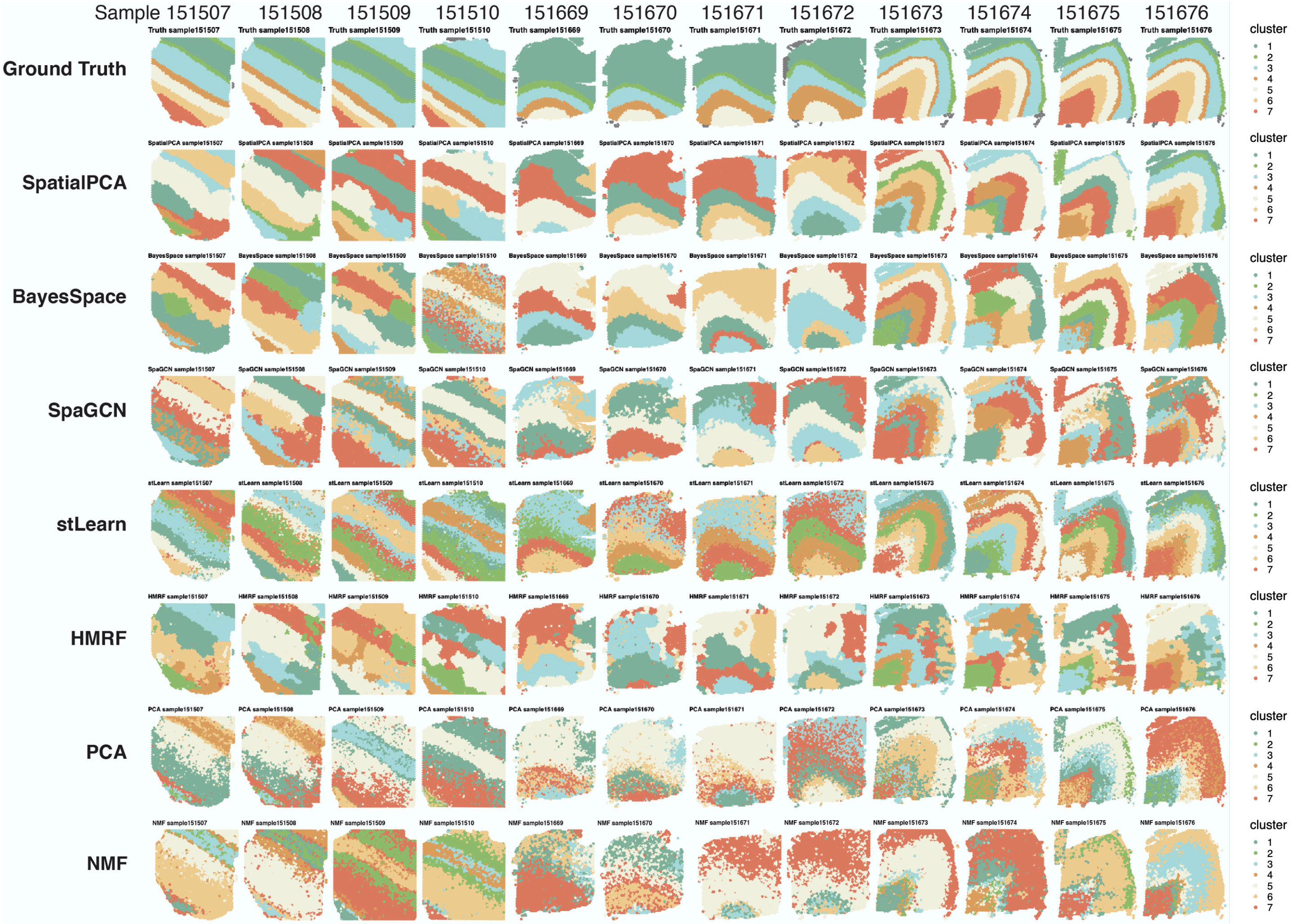

Supplementary Figure 12. Clustering results of different spatial domain detection methods in DLPFC across all twelve samples. First row: Ground truth annotation of all twelve samples**. Second row**: SpatialPCA clustering results. **Third row**: BayesSpace clustering results. **Fourth row**: SpaGCN clustering results. **Fifth row**: stLearn clustering results. **Sixth row**: HMRF clustering results. **Seventh row**: PCA clustering results. **Eighth row**: NMF clustering results. Clustering results of PCA and NMF are obtained with SVGs.

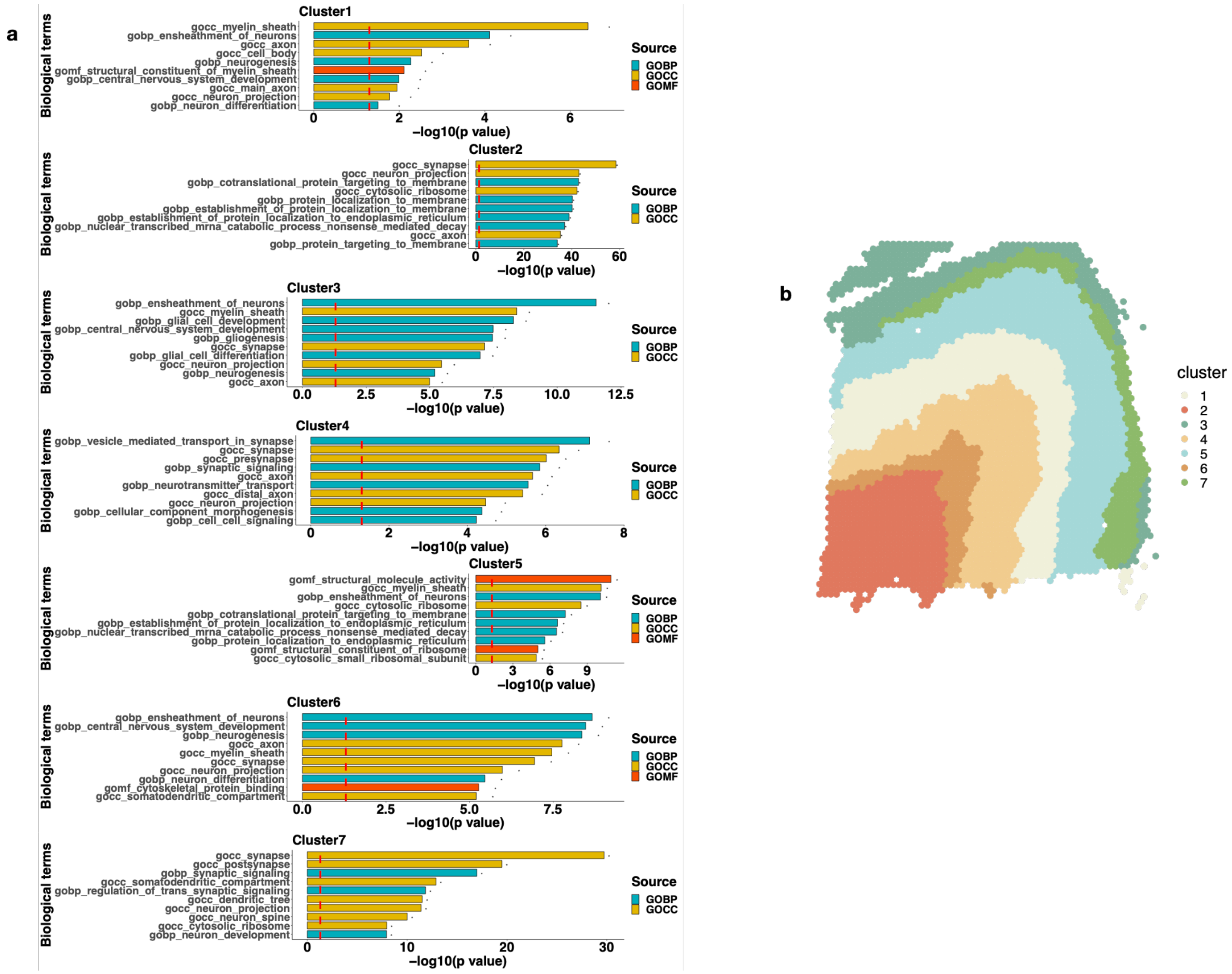

Supplementary Figure 13. Gene set enrichment analysis on the region-specific genes in the DLPFC data sample 151676. **a.** The top 10 enriched gene sets are shown for each of the seven detected tissue regions. Color represents different data sources for annotating the gene sets. The enrichment is given as -log10 adjusted p-value (g:SCS correction, details in Methods) of the region specific genes. The cluster annotations are shown in **b**.

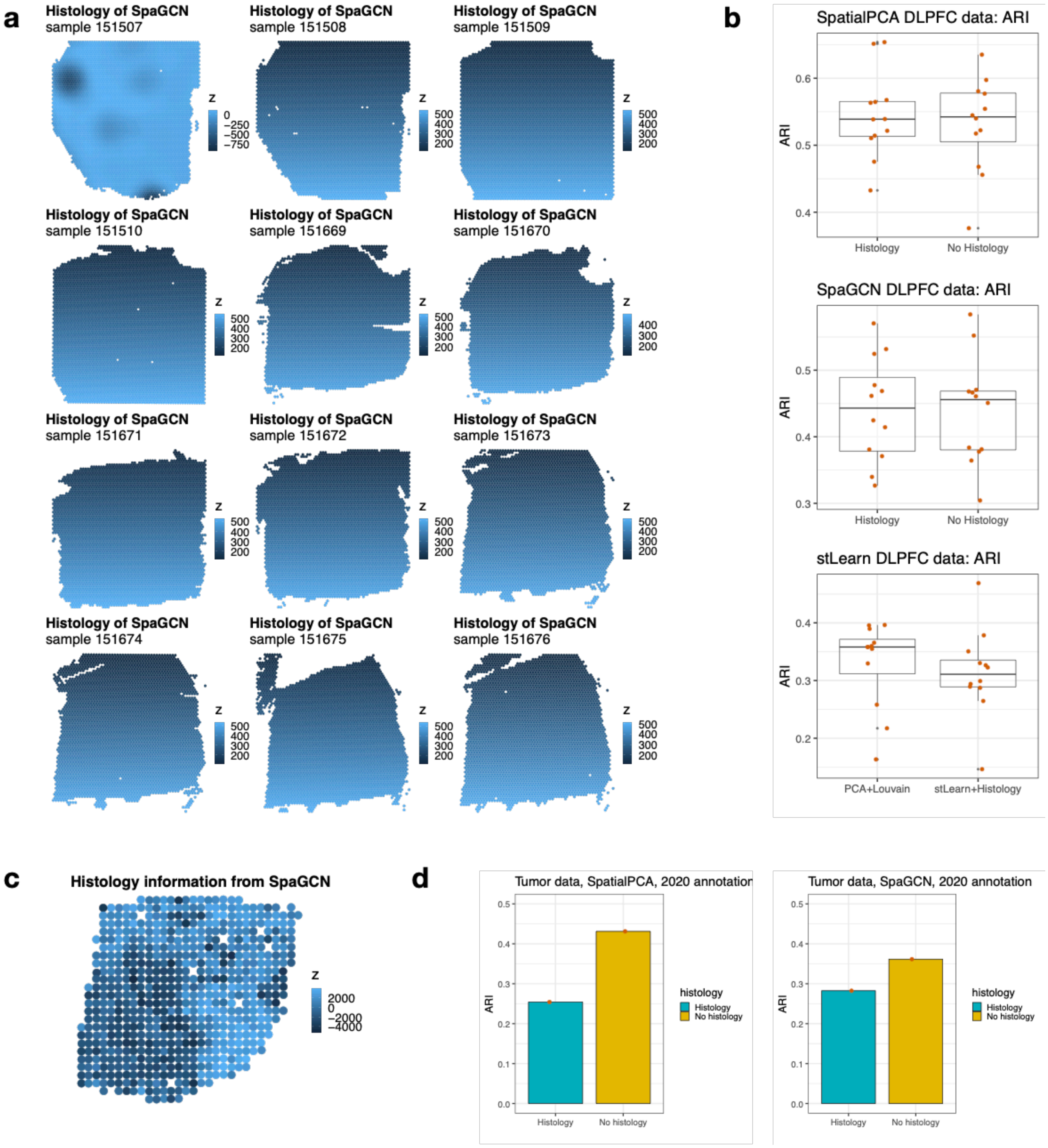

Supplementary Figure 14. Clustering results obtained with and without histology information in different methods in DLPFC data and ST tumor data. a. Visualization of the histology information extracted from the RGB values of the H&E image in DLPFC data through SpaGCN. **b**. Clustering results with and without histology information in SpatialPCA, SpaGCN, and stLearn in DLPFC data in all 12 sections. The adjusted Rand index (ARI, the higher the better) in stLearn was calculated based on SVGs. In the boxplot, the center line, box limits and whiskers denote the median, upper and lower quartiles, and 1.5× interquartile range, respectively. **c**. Visualization of the histology information extracted from the RGB values of the H&E image in ST tumor data through SpaGCN. **d**. Clustering results with and without histology information in SpatialPCA, SpaGCN, and stLearn in the ST tumor data (n=607 spots). The ground truth annotations are from the ST tumor data original paper (Andersson et al. 2020).

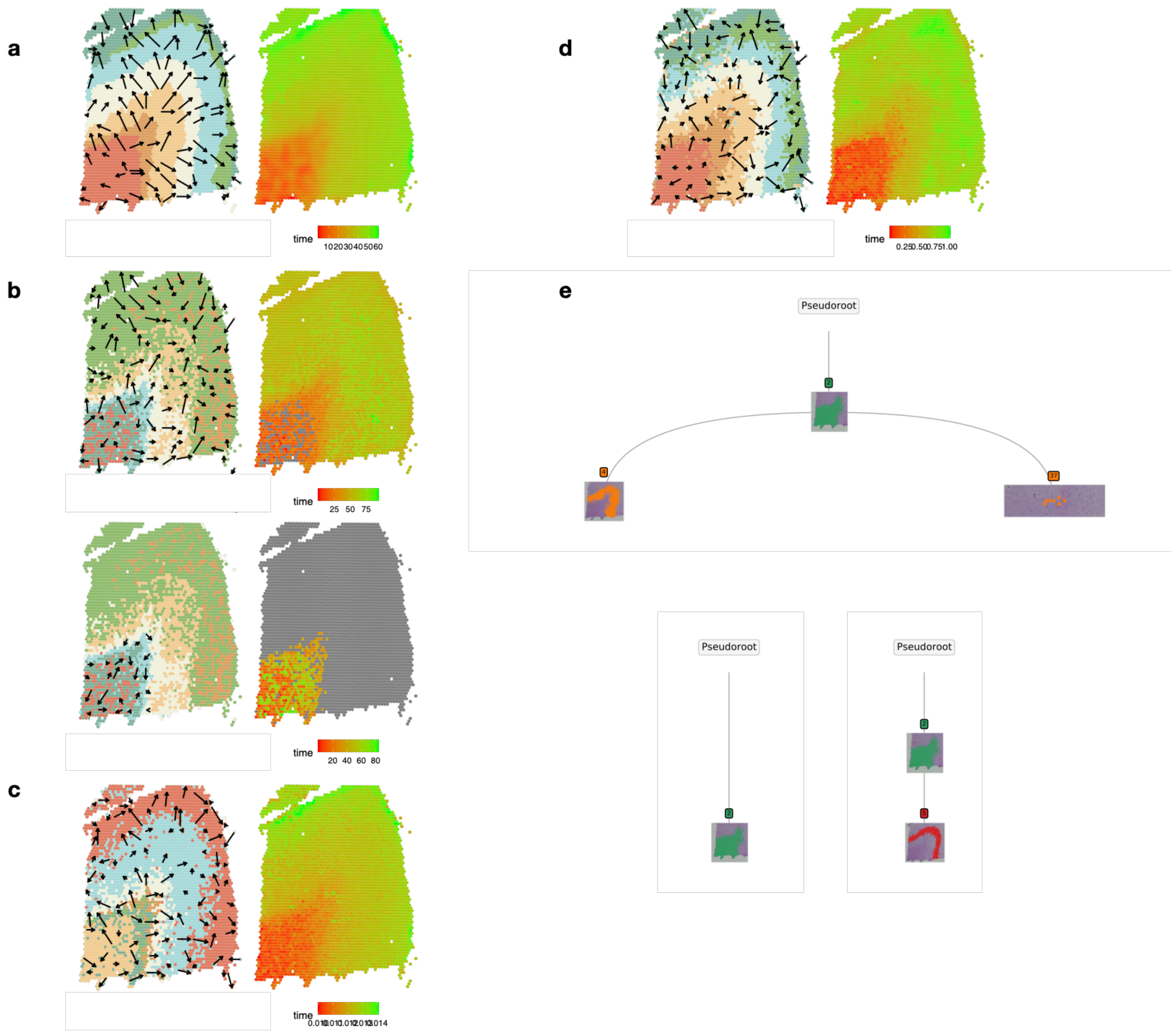

Supplementary Figure 15. Spatial trajectory inference results in the DLPFC data. **a.** Visualizaton of the trajectory inferred by SpatialPCA. Left: Arrows point from tissue locations with low pseudo-time to tissue locations with high pseudo-time. Color represents different tissue regions. Right: Visualization of pseudotime inferred from spatial PCs in SpatialPCA. **b-c.** Visualizaton of the trajectories inferred from PCs in PCA and NMF. **d.** Visualizaton of the pseudotime inferred by stLearn. We plotted the arrows in the same way as in SpatialPCA. **e.** Visualization of trajectories inferred by stLearn. The stLearn considers a pair of clusters at each time and find the order between clusters.

a. DLPFC

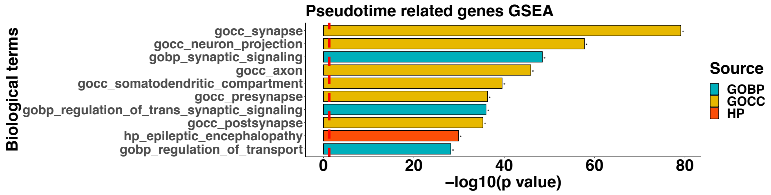

b. Slide-seq V2 cortical layers

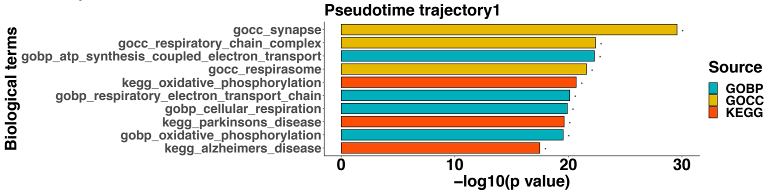

c. ST tumor

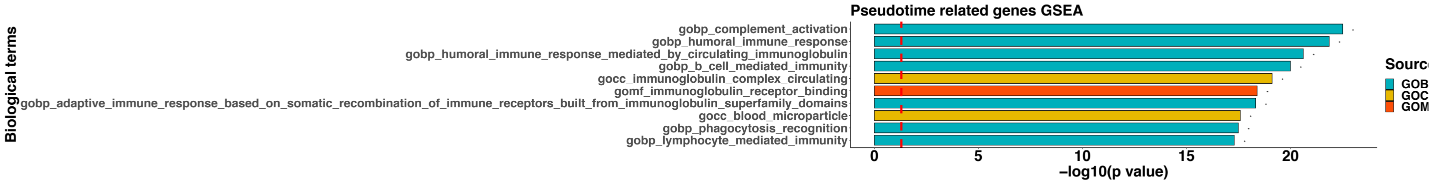

Supplementary Figure 16. Gene set enrichment analysis on the pseudo-time associated genes. The top 10 enriched gene sets are shown for each of the three detected trajectories. Color represents different data sources for annotating the gene sets. **a.** The enriched gene sets in DLPFC data. **b.** The enriched gene sets in cortical layers of the Slide-seq V2 data. **c.** The enriched gene sets in ST tumor data. The enrichment in **a**-**c** are given as -log10 adjusted p-value (g:SCS correction, details in Methods) of the genes associated with pseudo-time.

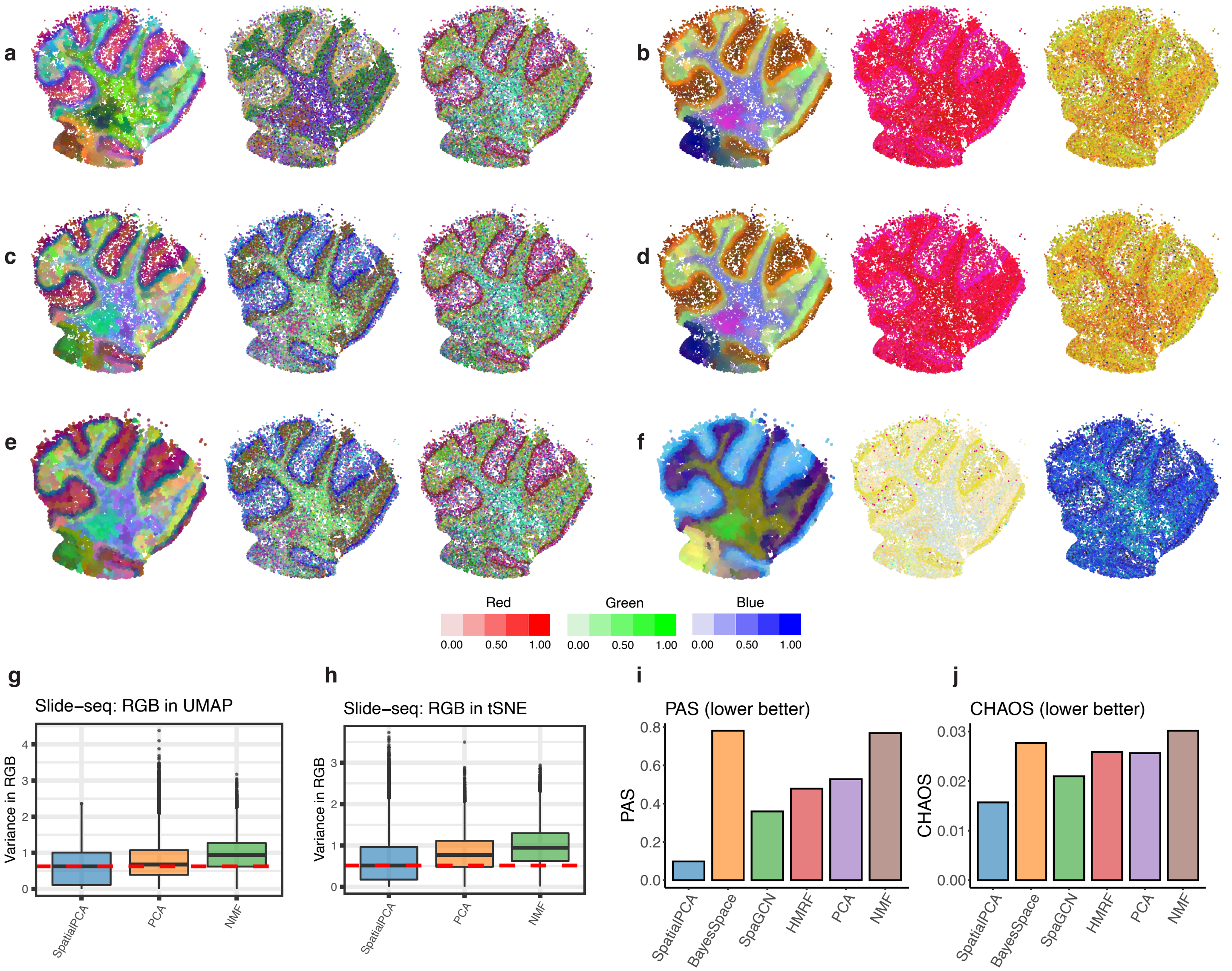

Supplementary Figure 17. RGB plots for the Slide-seq data. **a-f**. For SpatialPCA, PCA, and NMF, we summarized the inferred low dimensional components into three UMAP (**a, c, e**) or tSNE components (**b, d, f**) and visualized the three resulting components with red/green/blue (RGB) colors through the RGB plot. Color code corresponds to the RGB values of each location's three UMAP or tSNE components inferred from low dimensional components in dimension reduction. Different colors indicate different values for each of the three UMAP or tSNE components on the tissue section, highlighting the difference of the low dimensional components from different methods included in the panel. The RGB plot from SpatialPCA displays laminar organization of the cortex and show less color differences within a local area. We also scaled up spatial PCs/regular PCs 10 times (**c-d**) and 20 times (**e-f**) to see the influence of range of the PCs to RGB plots. The tSNE/UMAP results and RGB plots in figures (**c-f**) have very similar patterns as shown at the original scale (**a-b**). **g-h**. The weighted RGB values in SpatialPCA have lower variance than PCA or NMF in nearby spots (n=20,982 locations). The RGB plot from SpatialPCA displays tissue structure organization of the cerebellum and show less color differences within a local area. In the boxplots in **g-h**, the center line, box limits and whiskers denote the median, upper and lower quartiles, and 1.5× interquartile range, respectively. **i-j**. Spatial continuity of the inferred clusters as measured by percentage of abnormal spots (PAS, the lower the better) and spatial chaos score (CHAOS, the lower the better) in SpatialPCA is the lowest compared with BayesSpace, SpaGCN, HMRF, PCA and NMF.

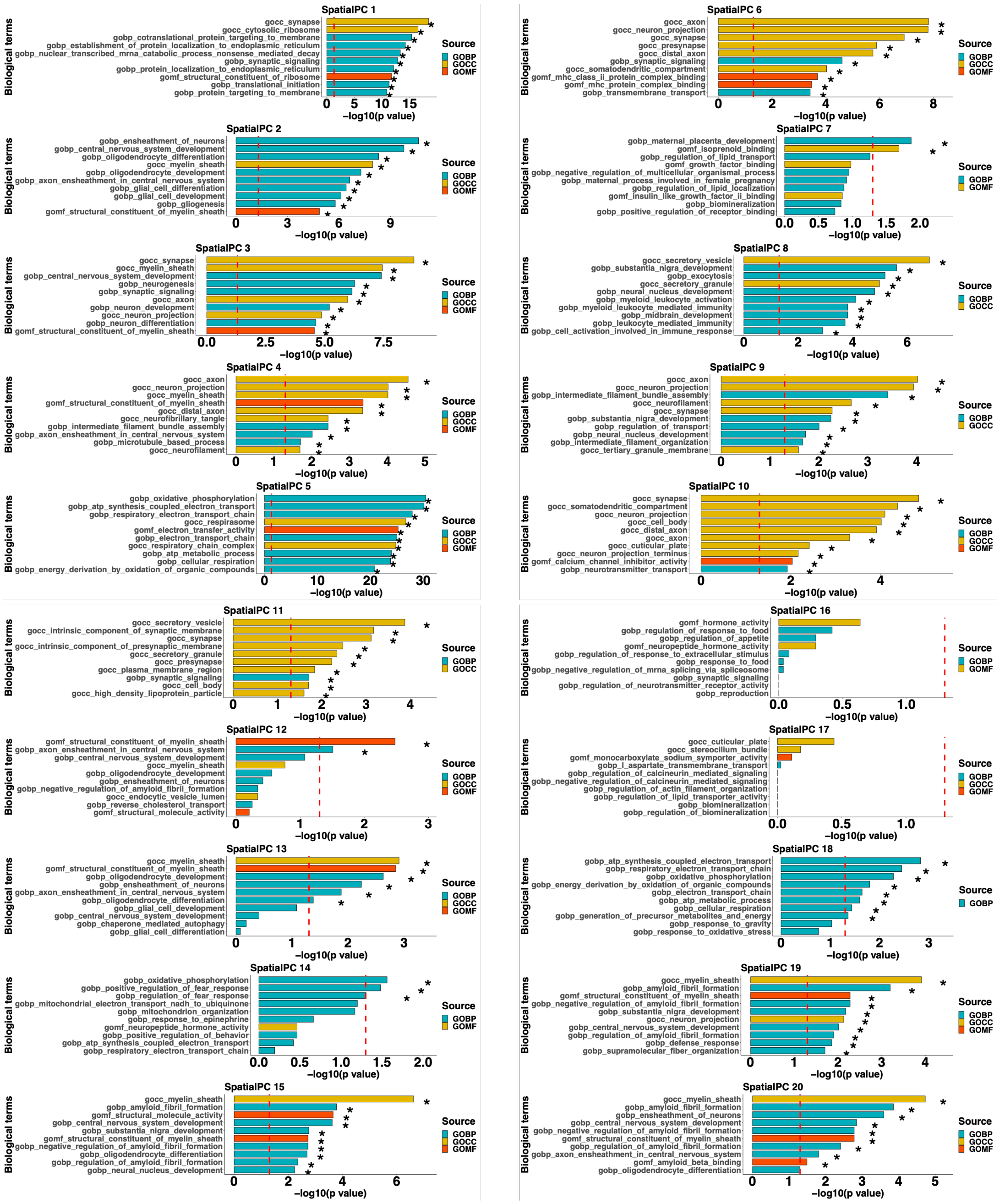

Supplementary Figure 18. Gene set enrichment analysis on the genes associated with Spatial PC values in the Slide-seq data. The top 10 enriched gene sets are shown for each of the 20 spatial PCs. Color represents different data sources for annotating the gene sets. The enrichment is given as -log10 adjusted p-value (g:SCS correction, details in Methods) of the genes associated with spatial PC values.

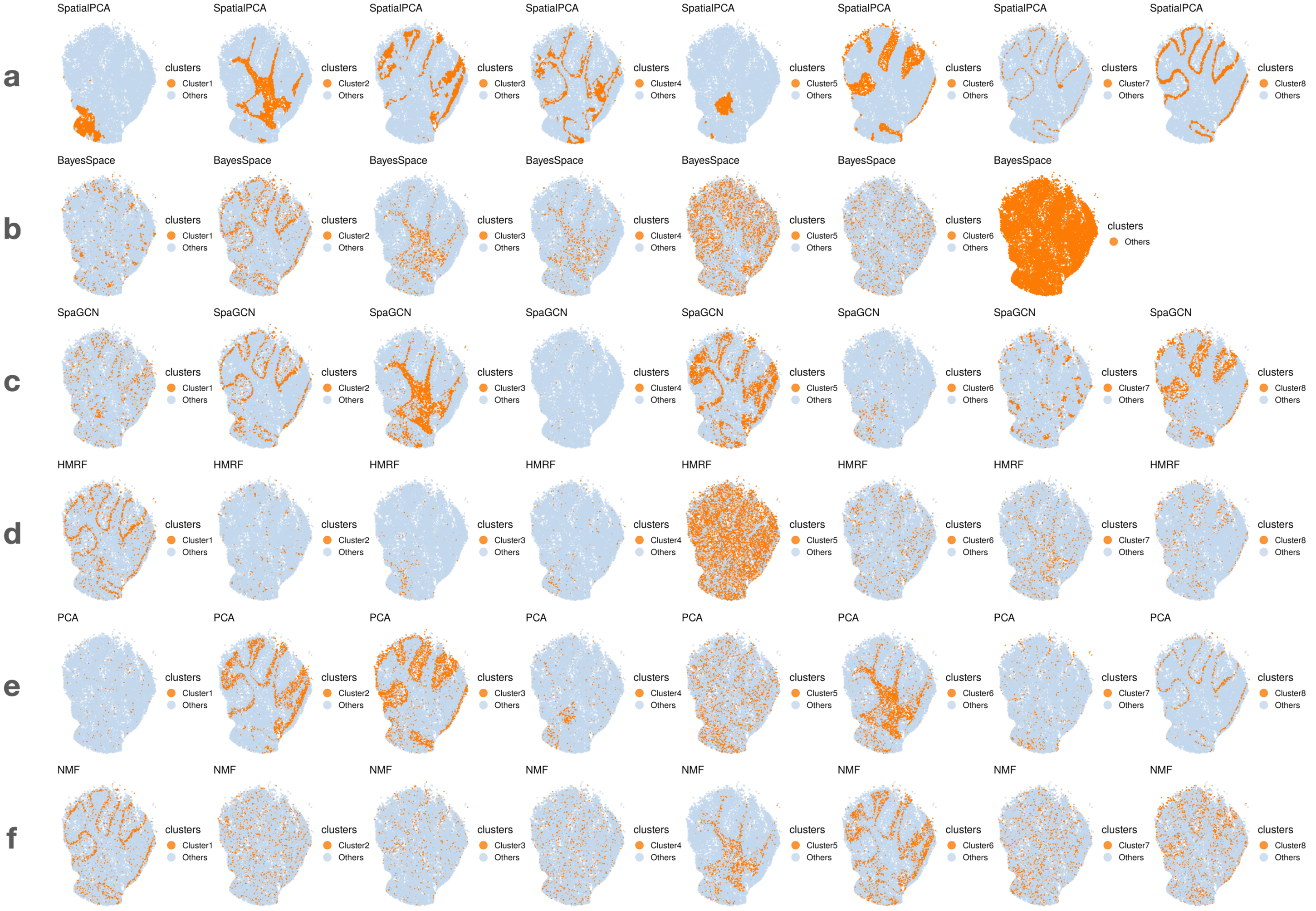

Supplementary Figure 19. Visualization of each spatial domain detected by different methods in the Slide-seq data. **a.** Visualization of the spatial domains detected by SpatialPCA. **b.** Visualization of the spatial domains detected by BayesSpace. **c.** Visualization of the spatial domains detected by SpaGCN. **d.** Visualization of the spatial domains detected by HMRF. **e.** Visualization of the spatial domains detected by PCA. **f.** Visualization of the spatial domains detected by NMF.

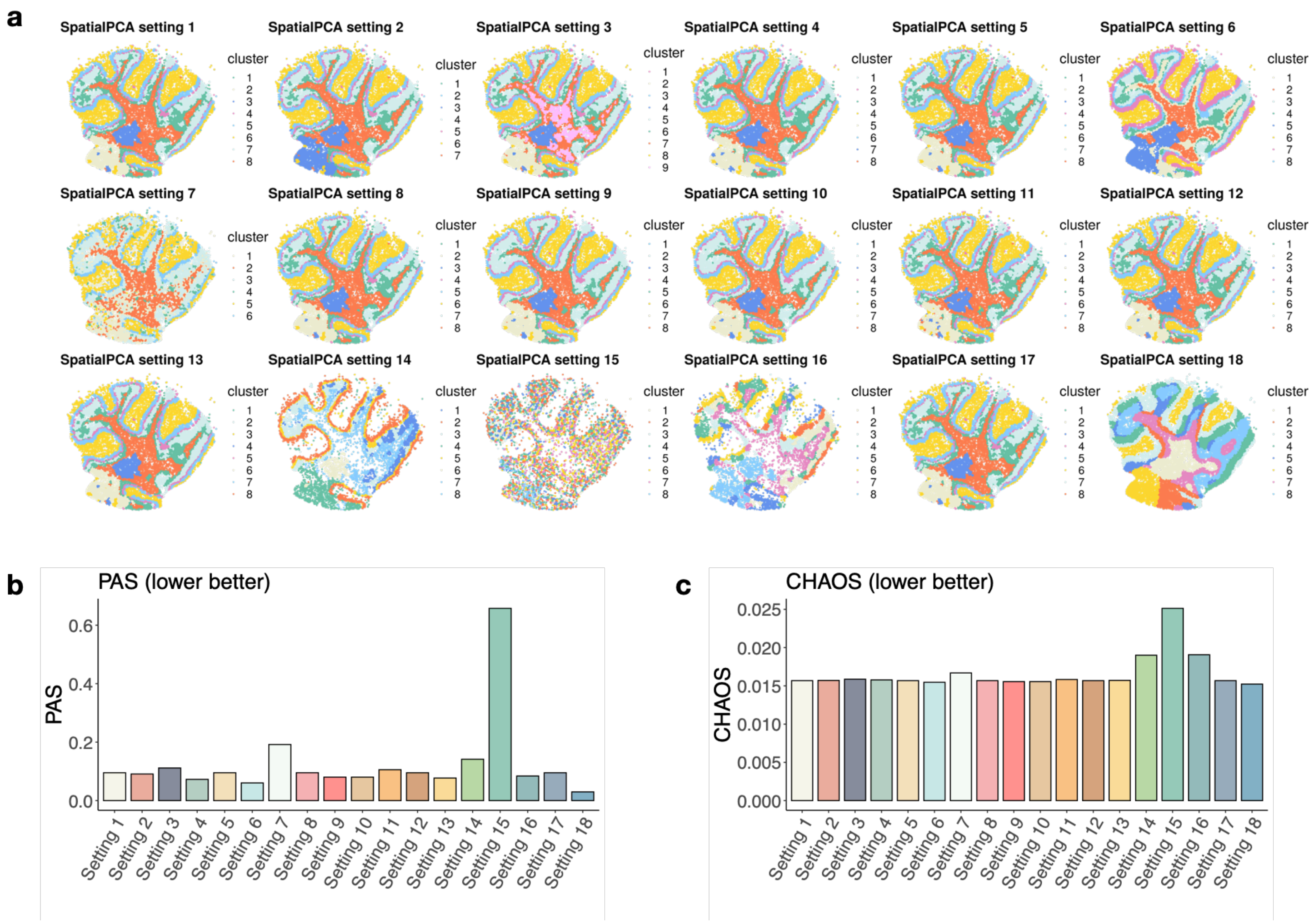

Supplementary Figure 20. Sensitivity analyses for the Slide-seq data. **a.** Clustering results from SpatialPCA are shown for different analytic settings. Setting 1: with 8 clusters. Setting 2: with 7 clusters. Setting 3: with 9 clusters. Setting 4: using top 500 spatially variable genes detected by SPARK-X. Setting 5: using top all spatially variable genes detected by SPARK-X. Setting 6: using all spatially variable genes detected by SPARK. Setting 7: using all highly variable genes detected by Seurat. Setting 8: using Gaussian kernel. Setting 9: using Cauchy kernel. Setting 10: using quadratic kernel. Setting 11: using top 10 Spatial PCs. Setting 12: using top 20 Spatial PCs. Setting 13: using top 30 Spatial PCs. Setting 14: controlling for cell types when selecting SVGs in SPARK-X. Setting 15: controlling for cell types in SpatialPCA. Setting 16: controlling for cell types by regressing them out from the input gene expression and take the residuals. Setting 15: controlling for cell density in the spots. Setting 17: gene expression normalized through SCTransform normalization. Setting 18: gene expression normalized through log normalization. **b.** Spatial continuity of the inferred clusters as measured by percentage of abnormal spots (PAS, the lower the better) for different settings. **c.** Spatial continuity of the inferred clusters as measured by spatial chaos score (CHAOS, the lower the better) for different settings.

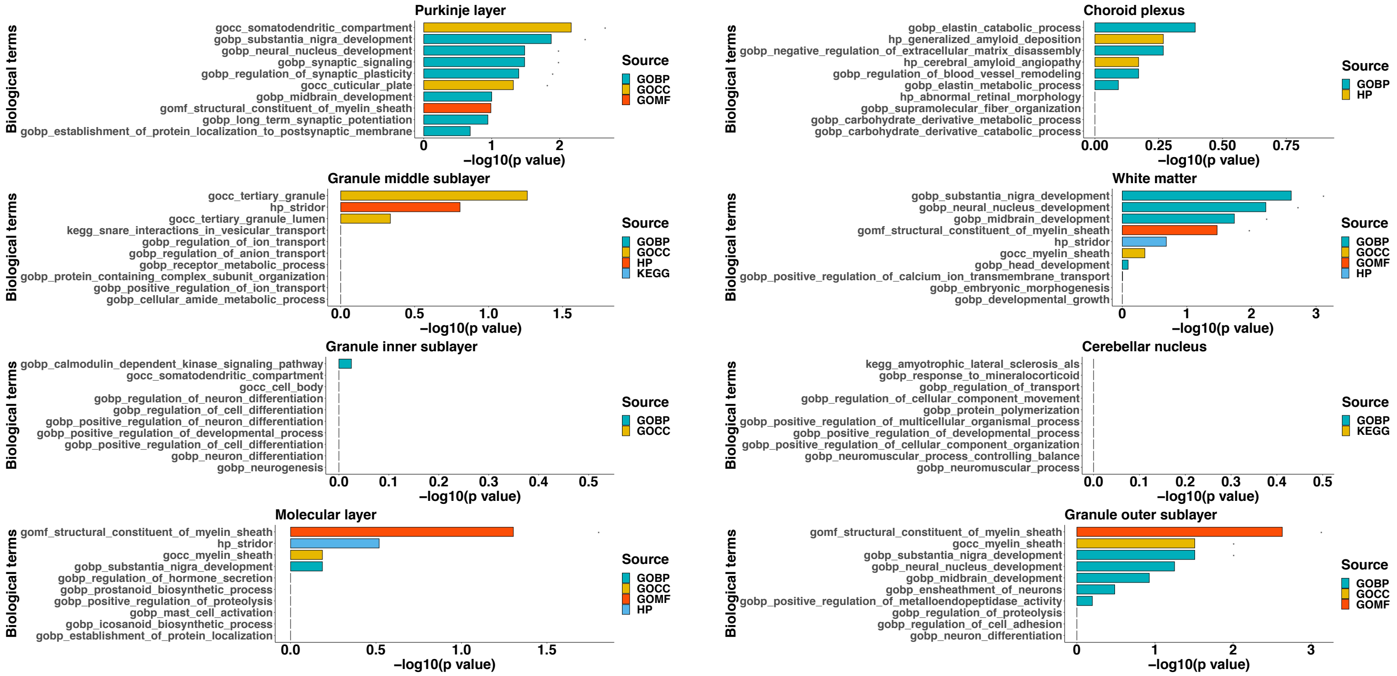

Supplementary Figure 21. Gene set enrichment analysis on the region-specific genes in the Slide-seq data. The top 10 enriched gene sets are shown for each of the eight detected tissue regions. Color represents different data sources for annotating the gene sets. The enrichment is given as -log10 adjusted p-value (g:SCS correction, details in Methods) of the region specific genes.

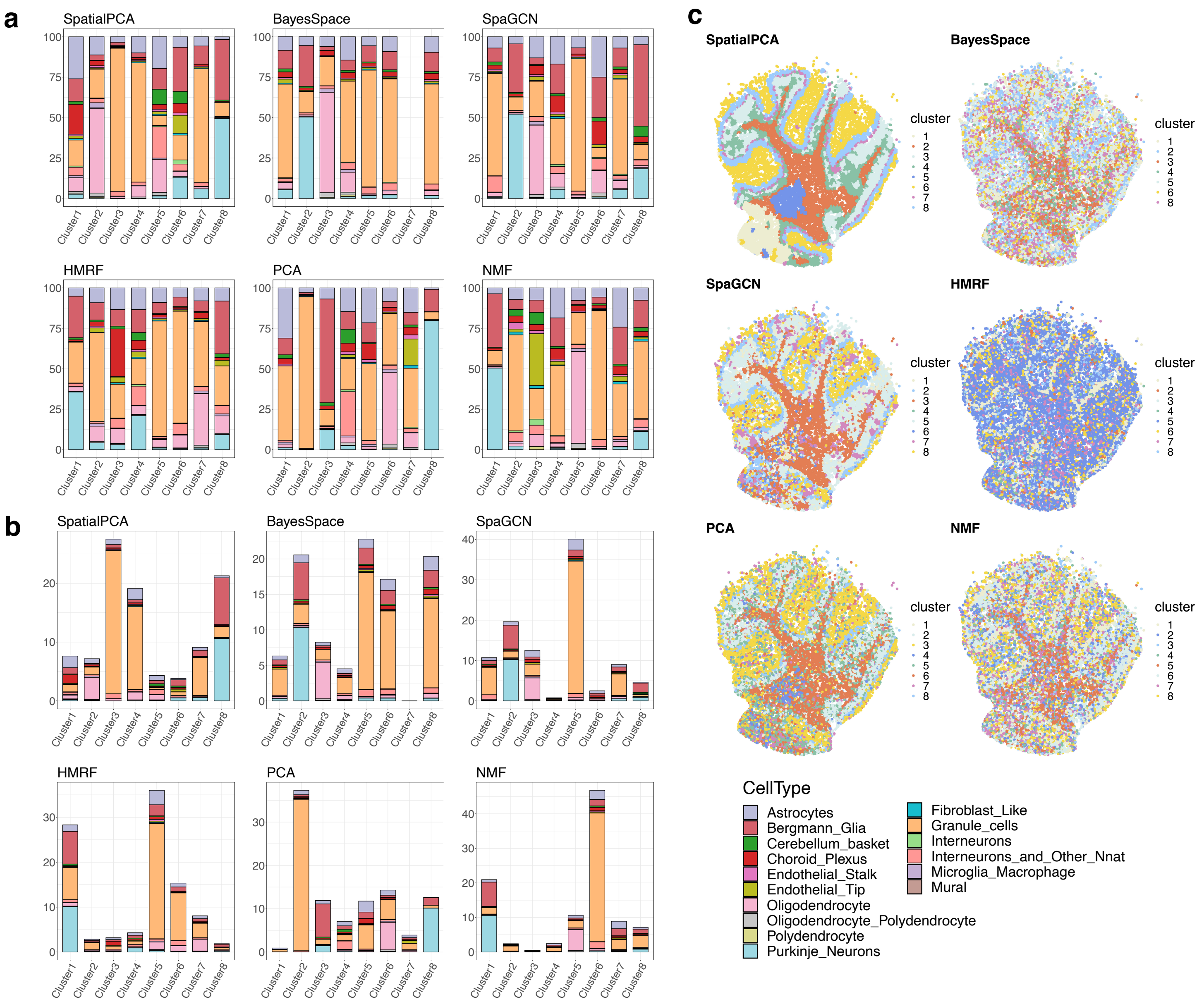

Supplementary Figure 22. Comparison of the cell type composition of the spatial domains detected by different methods in the Slide-seq data. The percentage of cell types annotated (y-axis) is shown on each tissue domain (x-axis) detected by different methods. Examined methods include SpatialPCA, SpaGCN, BayesSpace, HMRF, PCA, and NMF. **a.** Results are scaled with respect to each spatial domain, such that the summation of the cell type percentages in each domain is 100%. **b.** Results are scaled with respect to the cell types, such that the summation of all cell types across all tissue regions is 100%. **c.** Clustering results in each method. The clustering labels correspond to the x-axis in the left and middle panel.

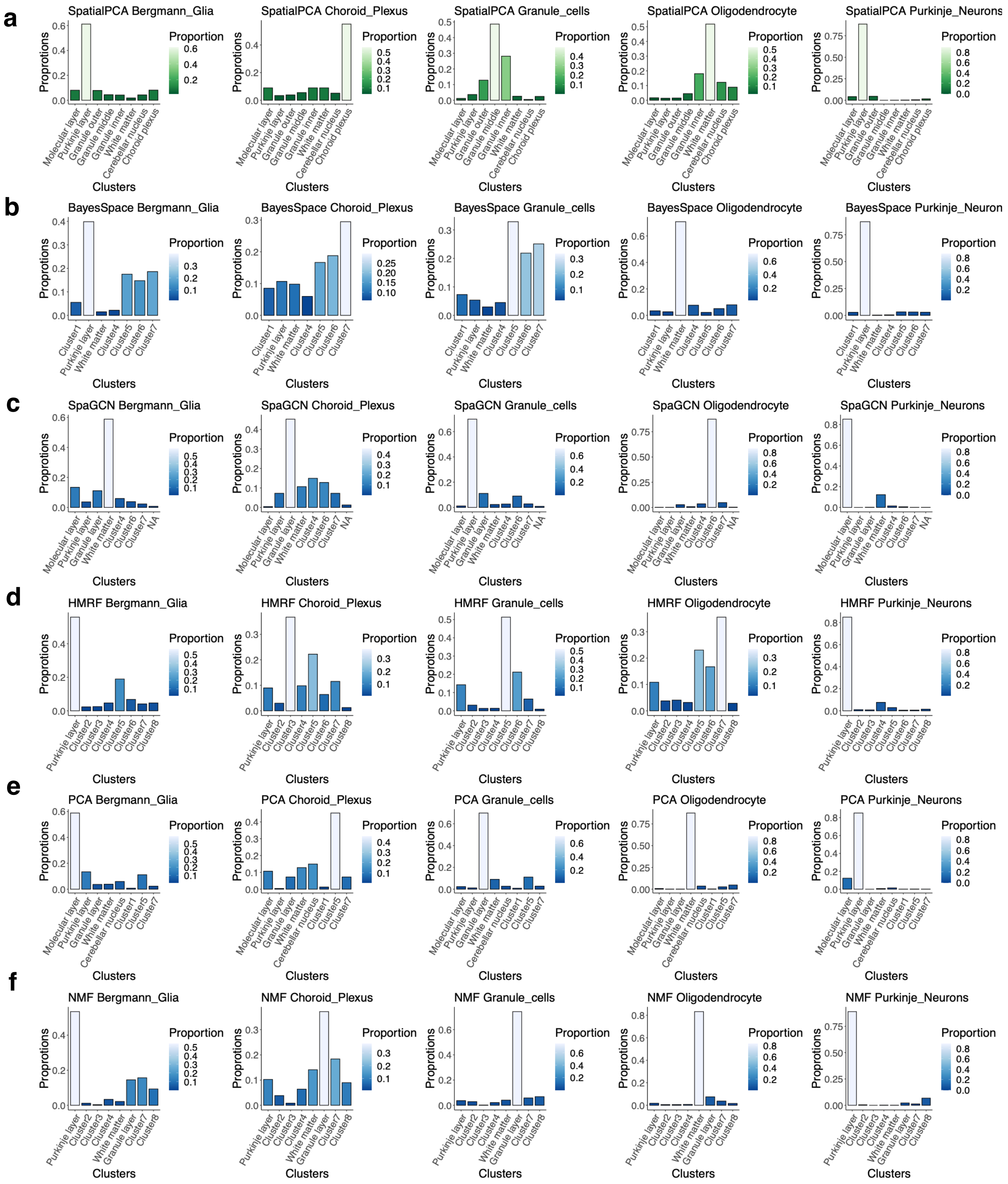

Supplementary Figure 23. Distribution of cell types in each cluster for different methods in the Slide-seq data. The summation of the cell type percentages in all clusters is 100% for SpatialPCA (**a**), BayesSpace (**b**), SpaGCN (**c**), HMRF (**d**), PCA (**e**) and NMF (**f**). The Bergmann glia cells and Purkinje neurons are located in the Purkinje layer, the choroid plexus cells are located in the choroid plexus, the granule cells are located in the granule cell layer, the oligodendrocyte cells are located in white matter.

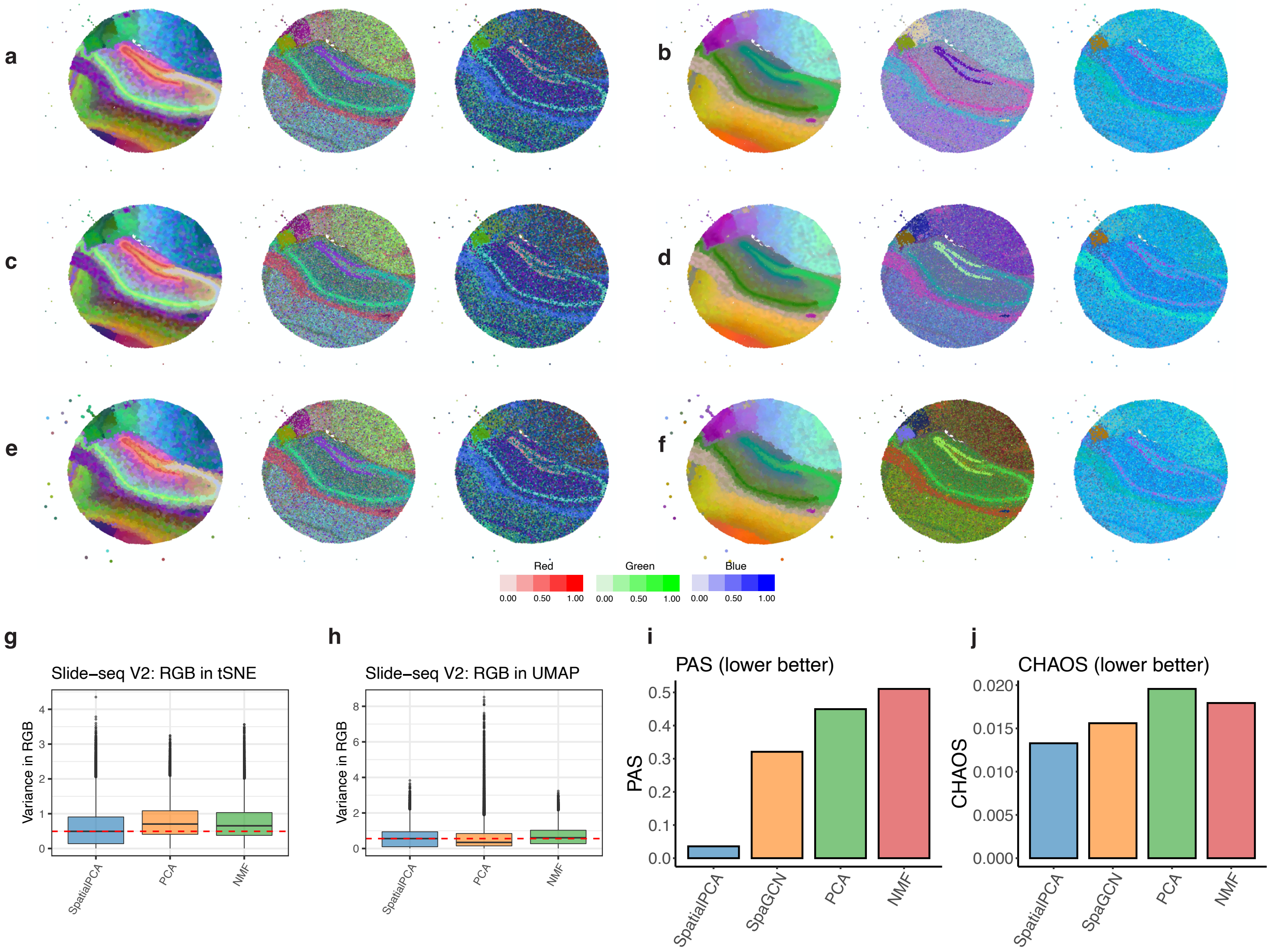

Supplementary Figure 24. RGB plots for the Slide-seq V2 data. **a-f.** For SpatialPCA, PCA, and NMF, we summarized the inferred low dimensional components into three tSNE (**a, c, e**) or UMAP components (**b, d, f**) and visualized the three resulting components with red/green/blue (RGB) colors through the RGB plot. Color code corresponds to the RGB values of each location's three UMAP or tSNE components inferred from low dimensional components in dimension reduction. Different colors indicate different values for each of the three UMAP or tSNE components on the tissue section, highlighting the difference of the low dimensional components from different methods included in the panel. The RGB plot from SpatialPCA displays tissue structure organization of the hippocampus region and show less color differences within a local area. We also scaled up spatial PCs/regular PCs 10 times (**c-d**) and 20 times (**e-f**) to see the influence of range of the PCs to RGB plots, the tSNE/UMAP results and RGB plots have very similar patterns as shown at the original scale (**a-b**). **g-h.** The weighted RGB values in SpatialPCA have lower variance than PCA or NMF in nearby spots (n=51,398 locations). In the boxplots in **g-h**, the center line, box limits and whiskers denote the median, upper and lower quartiles, and 1.5× interquartile range, respectively. **i-j.** Spatial continuity of the inferred clusters as measured by percentage of abnormal spots (PAS, the lower the better) and spatial chaos score (CHAOS, the lower the better) in SpatialPCA is the lowest compared with BayesSpace, SpaGCN, PCA and NMF.

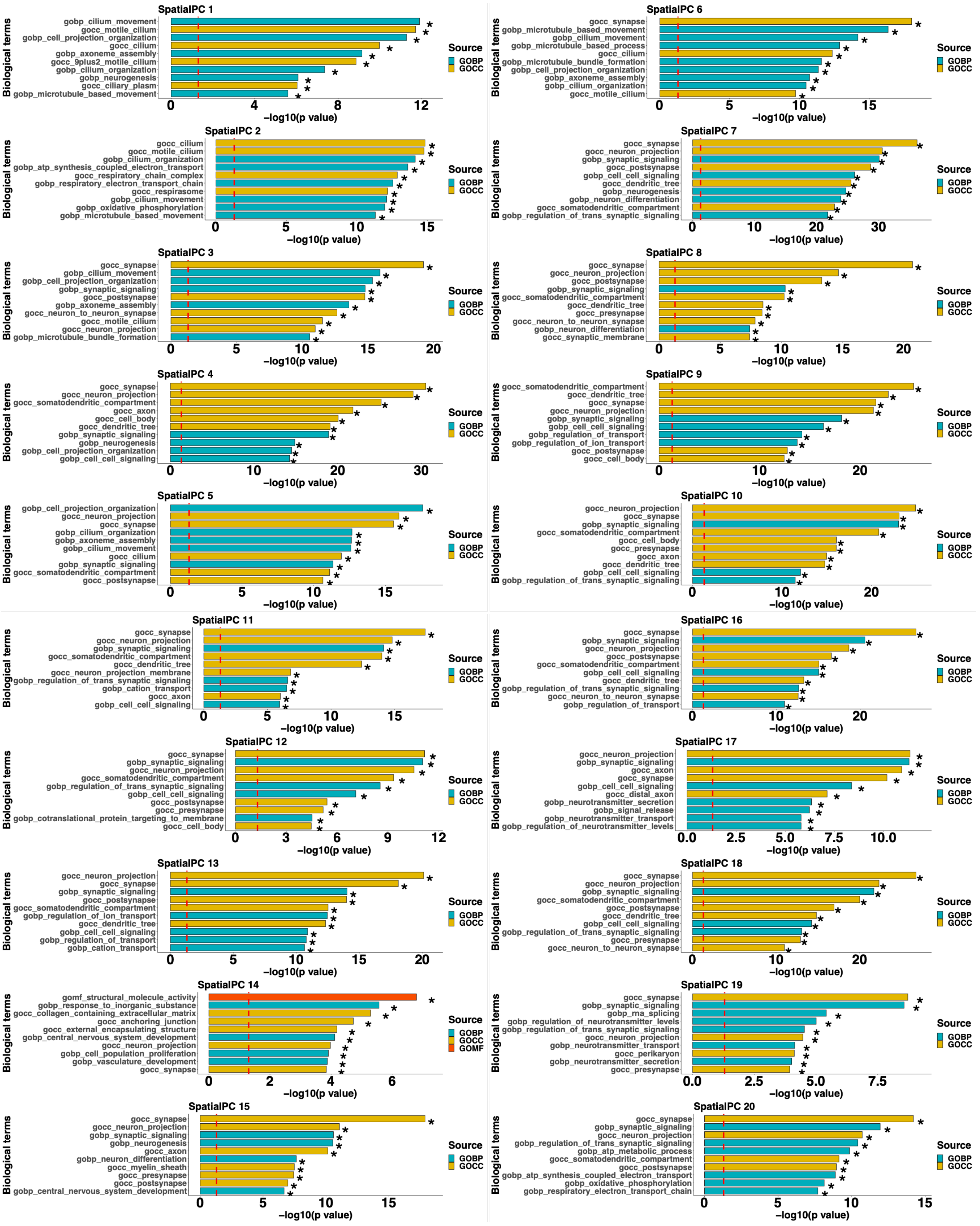

Supplementary Figure 25. Gene set enrichment analysis on the genes associated with Spatial PC values in the Slide-seq V2 data. The top 10 enriched gene sets are shown for each of the 20 spatial PCs. Color represents different data sources for annotating the gene sets. The enrichment is given as -log10 adjusted p-value (g:SCS correction, details in Methods) of the genes associated with spatial PC values.

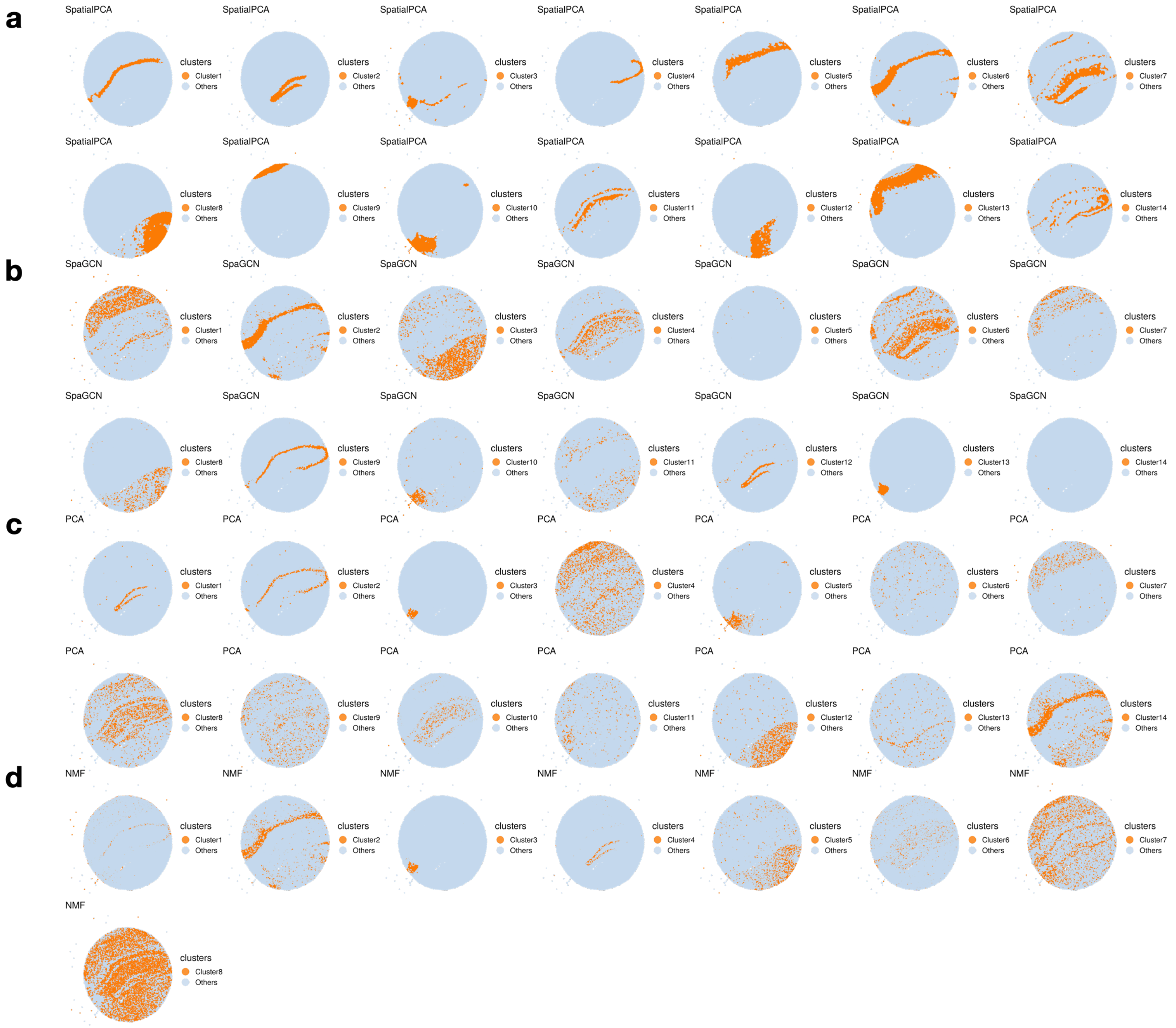

Supplementary Figure 26. Visualization of each spatial domain detected by each method separately in Slide-seq V2 data. **a.** Visualization of the spatial domains detected by SpatialPCA. **b.** Visualization of the spatial domains detected by SpaGCN. **c.** Visualization of the spatial domains detected by PCA. **d.** Visualization of the spatial domains detected by NMF.

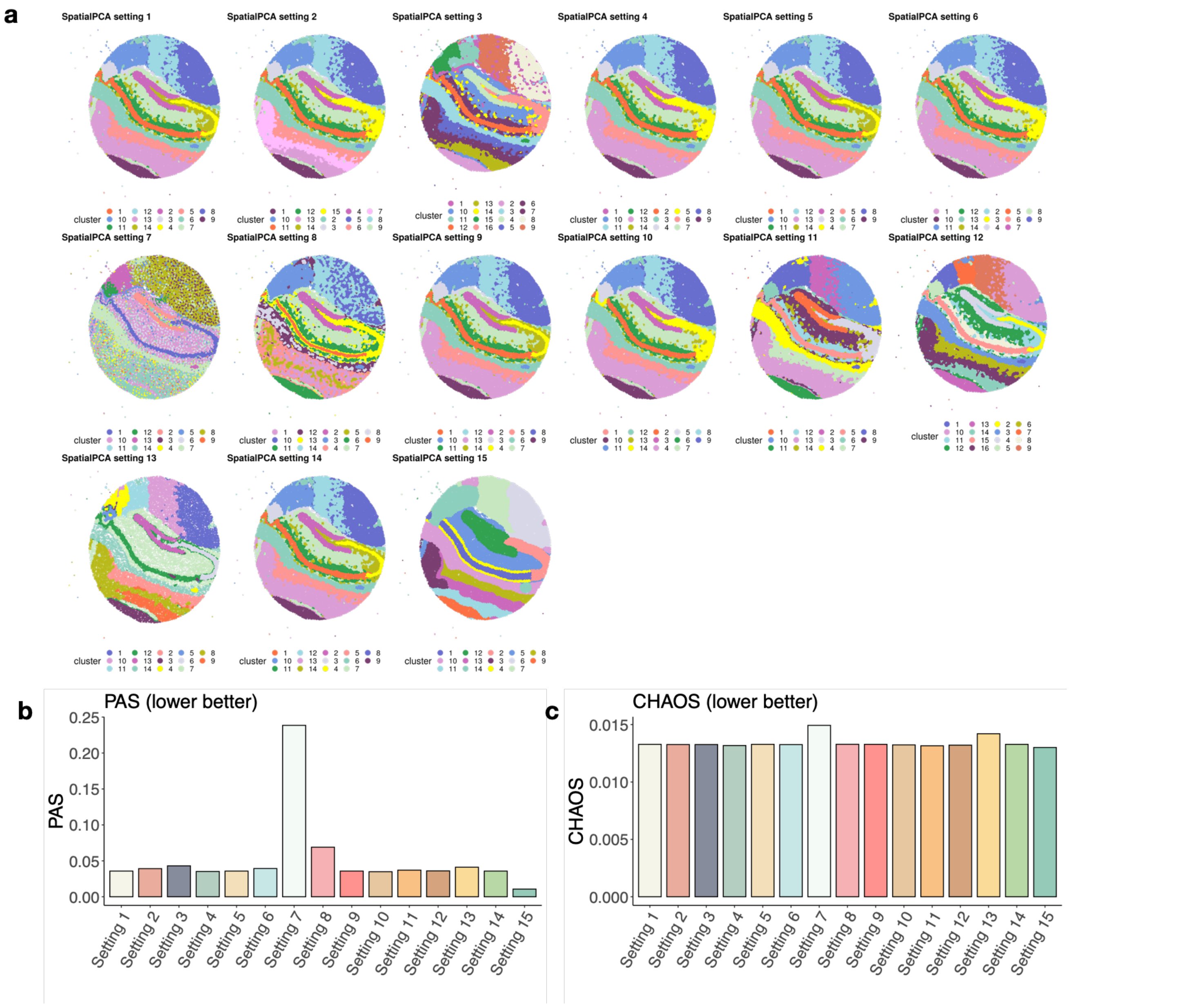

Supplementary Figure 27. Sensitivity analyses in the Slide-seq V2 data. **a.** Setting 1: clustering results obtained with 14 clusters. Setting 2: clustering results obtained with 15 clusters. Setting 3: clustering results obtained with 13 clusters. Setting 4: clustering results obtained using top 2000 spatially variable genes detected by SPARK-X. Setting 5: clustering results obtained using top 3000 spatially variable genes detected by SPARK-X. Setting 6: clustering results obtained using top 4000 spatially variable genes detected by SPARK-X. Setting 7: clustering results obtained using all highly variable genes detected by Seurat. Setting 8: clustering results obtained using top 10 Spatial PCs. Setting 9: clustering results obtained using top 20 Spatial PCs. Setting 10: clustering results obtained using top 30 Spatial PCs. Setting 11: clustering results obtained by controlling cell types in SpatialPCA. Setting 12: clustering results obtained through controlling cell types by regressing them out from the input gene expression and take the residuals. Setting 13: clustering results obtained by controlling for cell density in the spots. Setting 14: clustering results obtained by SpatialPCA with gene expression normalized through SCTransform normalization. Setting 15: clustering results obtained by SpatialPCA with gene expression normalized through log normalization. **b.** Clustering spatial continuity measured by percentage of abnormal spots (PAS, the lower the better) in different settings. **c.** Clustering spatial continuity measured by spatial chaos score (CHAOS, the lower the better) in different settings.

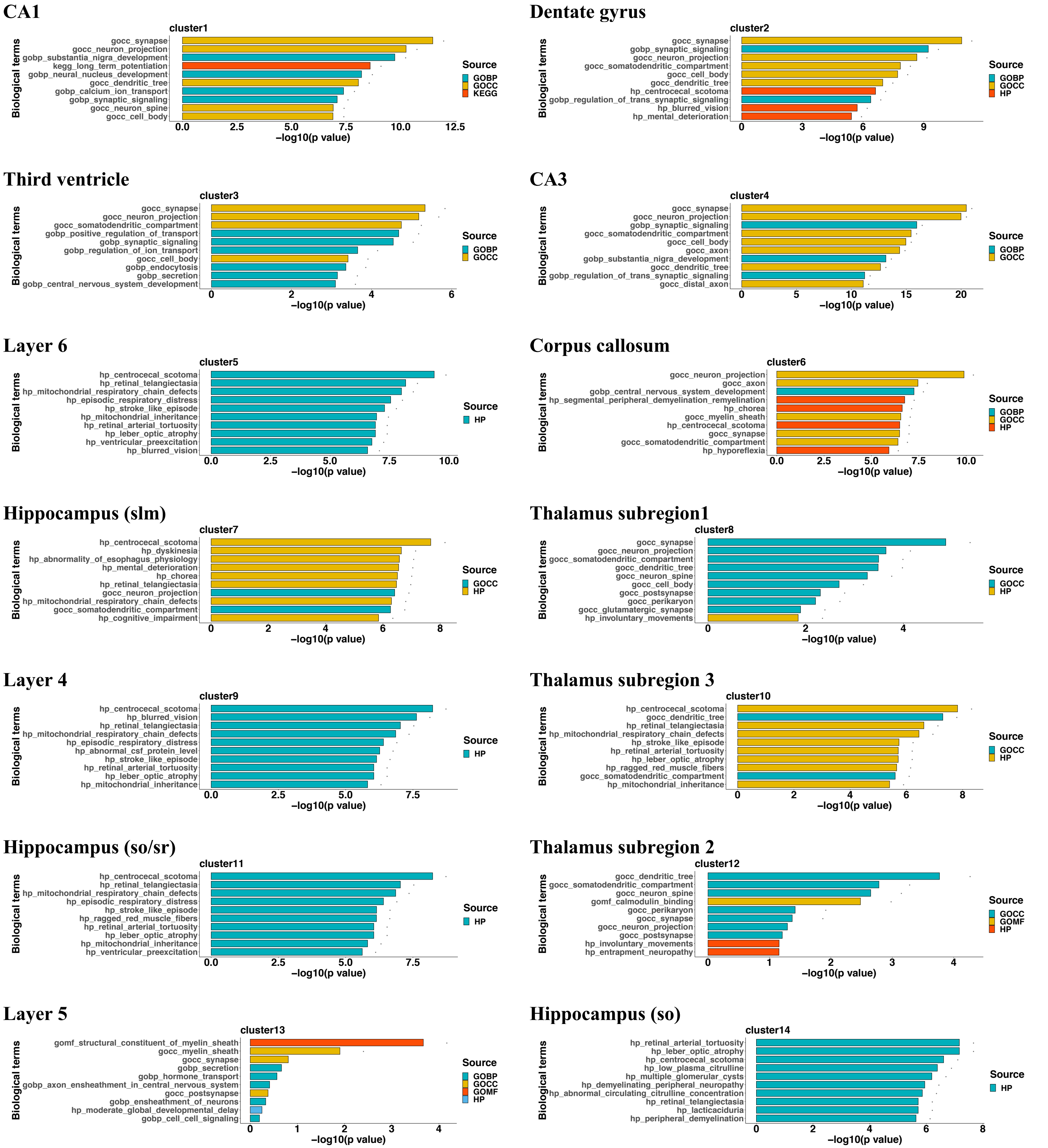

Supplementary Figure 28. Gene set enrichment analysis on the region-specific genes in the Slide-seq V2 data. The top 10 enriched gene sets are shown for each of the eight detected tissue regions. Color represents different data sources for annotating the gene sets. The enrichment is given as -log10 adjusted p-value (g:SCS correction, details in Methods) of the region specific genes.

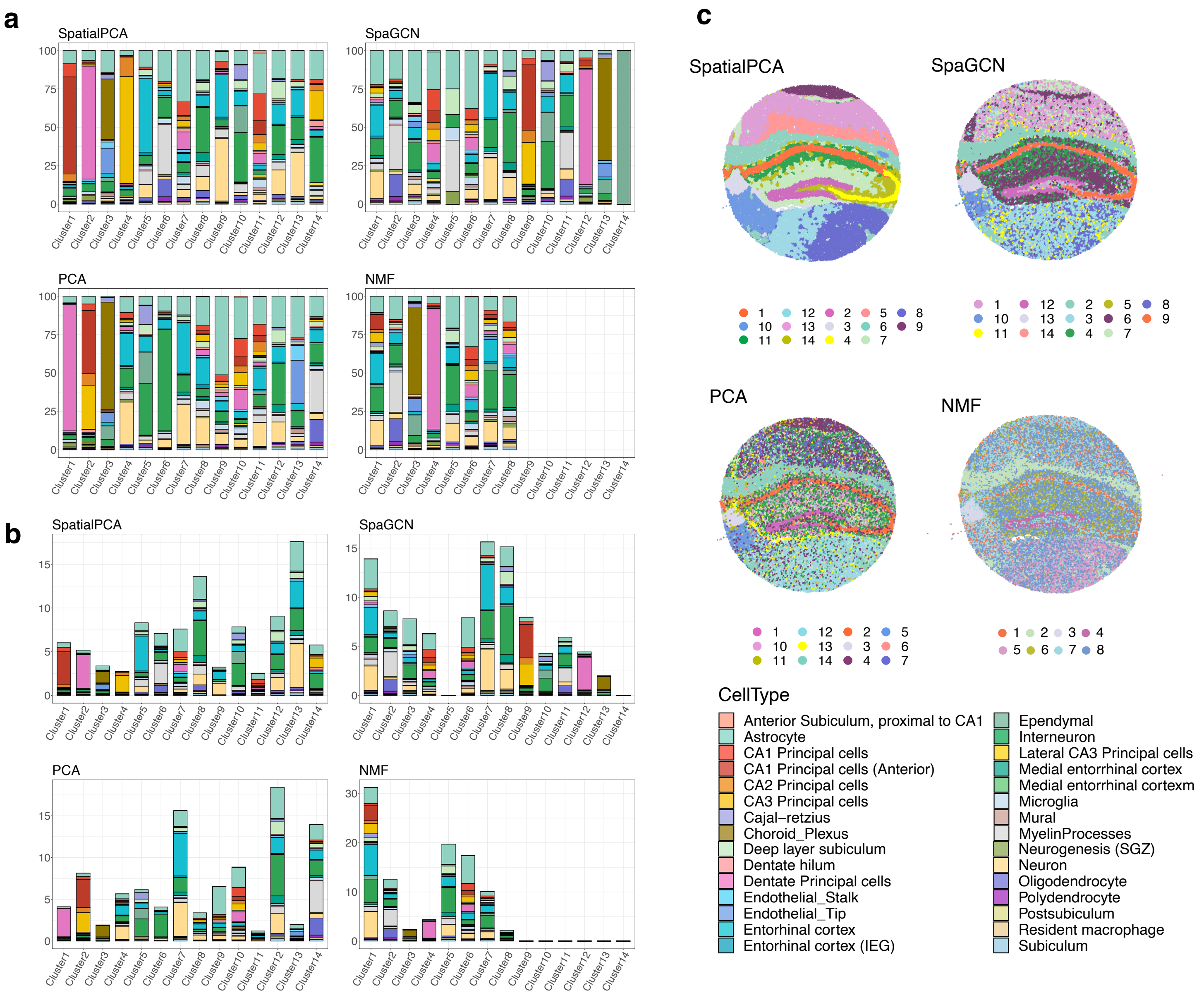

Supplementary Figure 29. Comparison of the cell type composition of the spatial domains detected by different methods in the Slide-seq V2 data. The percentage of cell types annotated (y-axis) is shown on each tissue domain (x-axis) detected by different methods. Examined methods include SpatialPCA, SpaGCN, PCA, and NMF. **a.** results are scaled with respect to each spatial domain, such that the summation of the cell type percentages in each domain is 100%. **b.** results are scaled with respect to the cell types, such that the summation of all cell types across all tissue regions is 100%. **c.** Clustering results in each method. The clustering labels correspond to the x-axis in **b**.

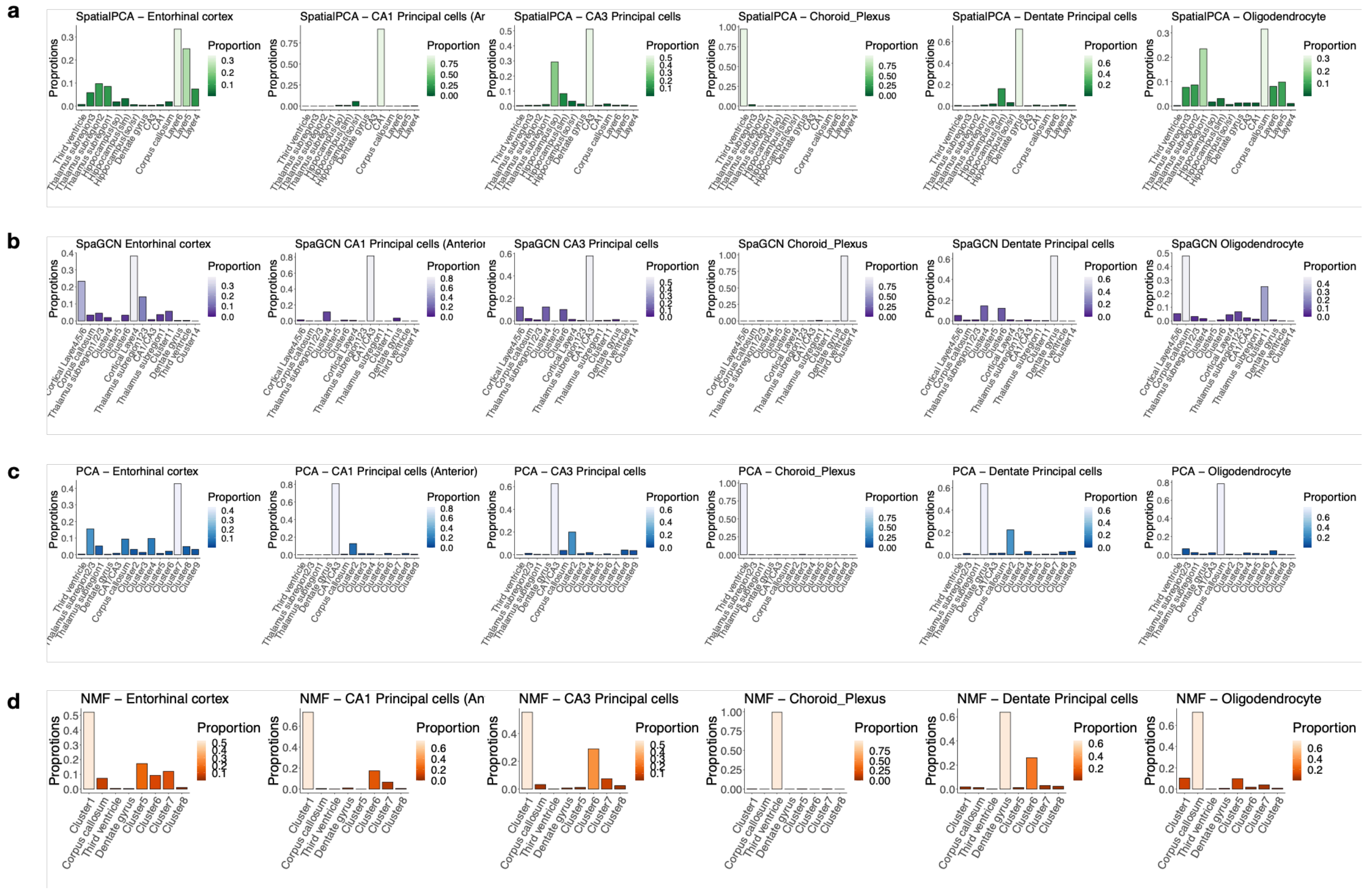

Supplementary Figure 30. Distribution of cell types in each cluster for different methods in Slide-seq V2 data. The summation of the cell type percentages in all clusters is 100% for SpatialPCA (**a**), SpaGCN (**b**), PCA (**c**) and NMF (**d**). The entorhinal cortex cells are located in the cortical layers 4-6; the CA1 principal cells (anterior) are located in the CA1 region; the CA3 principal cells are located in the CA3 region; the choroid plexus are located in the third ventricle; the dentate principle cells are located in the dentate gyrus; and the oligodendrocyte are located in the corpus callosum as detected by SpatialPCA.

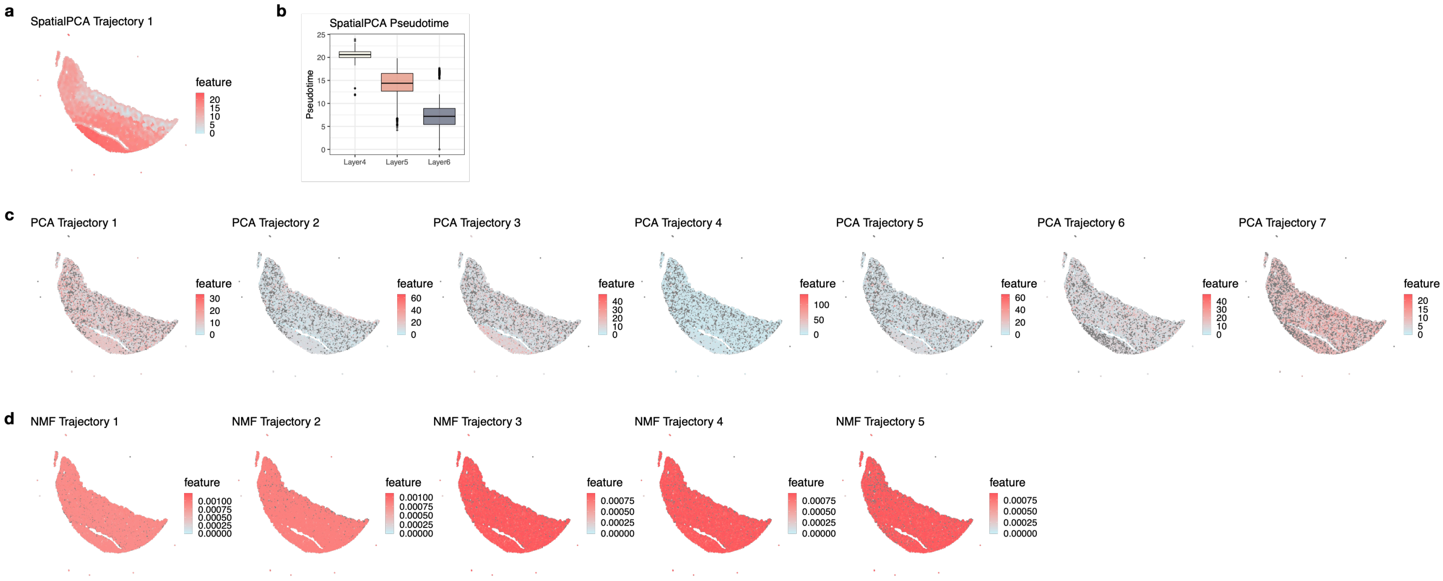

Supplementary Figure 31. Spatial trajectory inference results in the cortical layers of the Slide-seq V2 data. **a.** Visualizaton of the pseudo-time for the inferred trajectory in cortical layers of the Slide-seq V2 data in SpatialPCA. **b.** Boxplot showing the pseudotime of locations inferred by SpatialPCA in cortical layer 4, 5, and 6 (n=13,195 locations). In the boxplot, the center line, box limits and whiskers denote the median, upper and lower quartiles, and 1.5× interquartile range, respectively. **c.** Visualizaton of the inferred pseudo-time for seven trajectories (from left to right) in cortical layers of the Slide-seq V2 data in PCA. **d.** Visualizaton of the inferred pseudo-time for five trajectories (from left to right) in cortical layers of the Slide-seq V2 data in NMF.

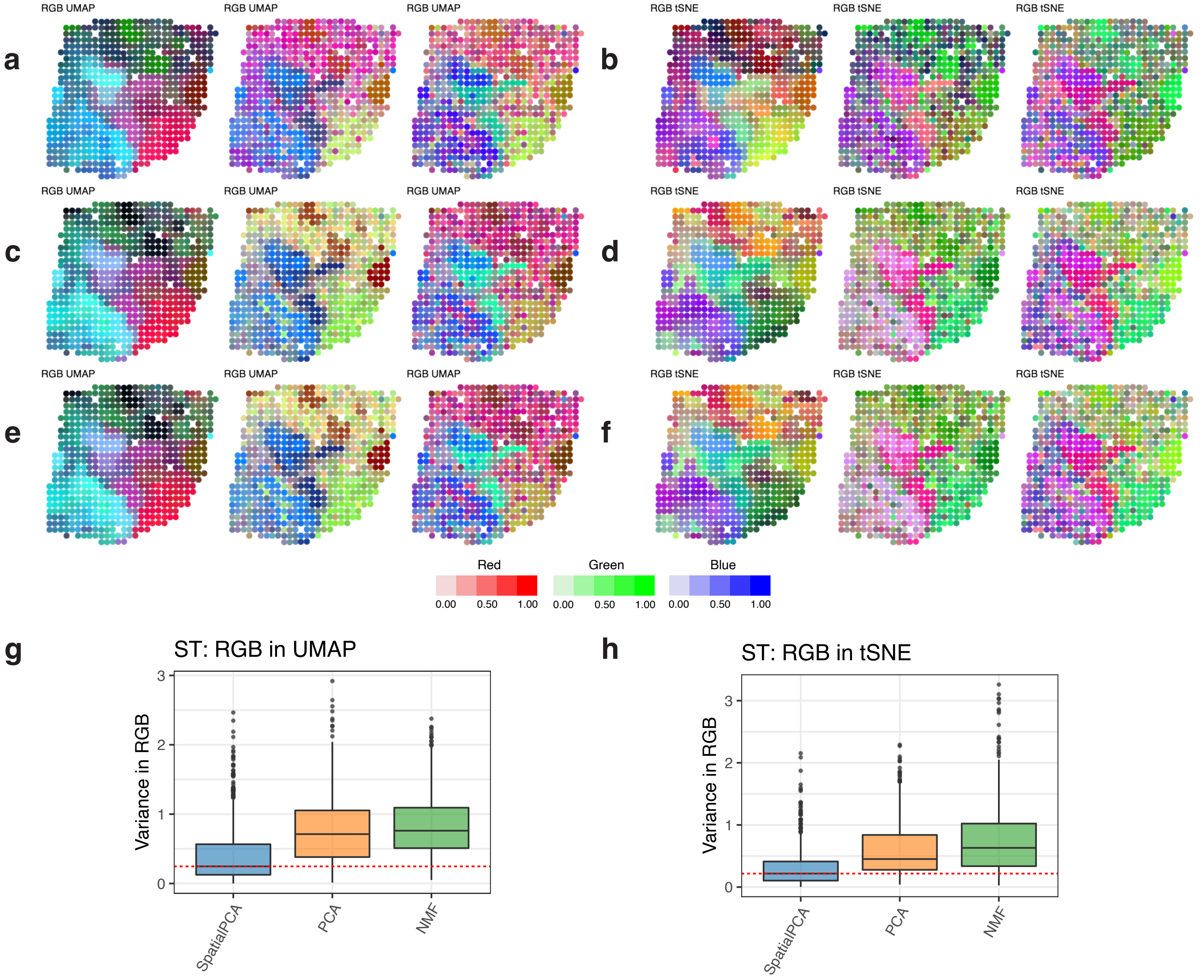

Supplementary Figure 32. RGB plots for the ST data. **a-f**. For SpatialPCA, PCA, and NMF, we summarized the inferred low dimensional components into three UMAP components (**a, c, e**) and tSNE (**b, d, f**) and visualized the three resulting components with red/green/blue (RGB) colors through the RGB plot. Color code corresponds to the RGB values of each location's three UMAP or tSNE components inferred from low dimensional components in dimension reduction. Different colors indicate different values for each of the three UMAP or tSNE components on the tissue section, highlighting the difference of the low dimensional components from different methods included in the panel. The RGB plot from SpatialPCA displays tissue structure organization of the breast tumor and show less color differences within a local area. We also scaled up spatial PCs/regular PCs 10 times (**c-d**) and 20 times (**e-f**) to see the influence of range of the PCs to RGB plots, the tSNE/UMAP results and RGB plots have very similar patterns as shown at the original scale (**a-b**). **g-h**. The weighted RGB values in SpatialPCA have lower variance than PCA or NMF in nearby spots (n=607 spots). In the boxplots in **g-h**, the center line, box limits and whiskers denote the median, upper and lower quartiles, and 1.5× interquartile range, respectively.

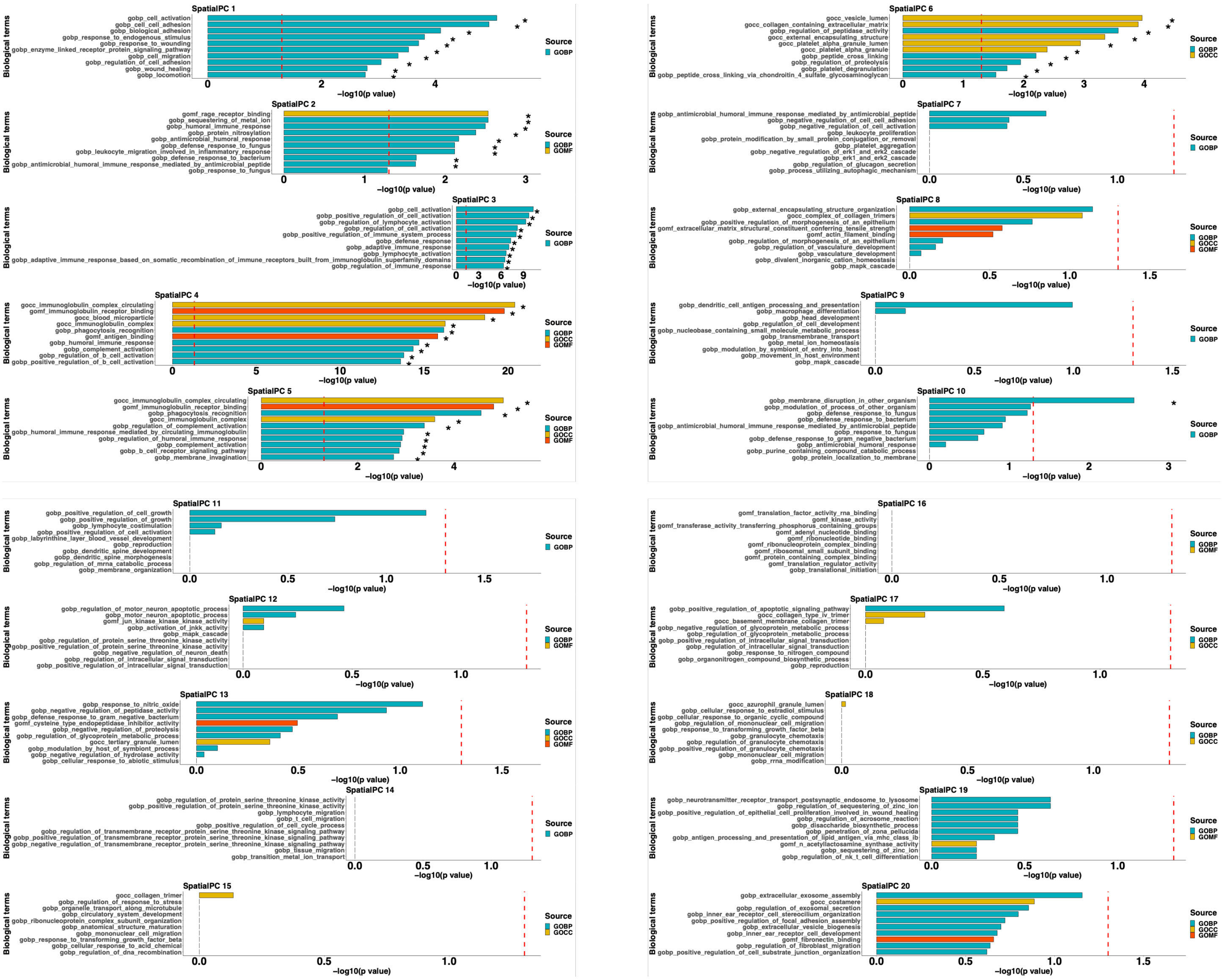

Supplementary Figure 33. Gene set enrichment analysis on the genes associated with Spatial PC values in the ST tumor data. The top 10 enriched gene sets are shown for each of the 20 spatial PCs. Color represents different data sources for annotating the gene sets. The enrichment is given as -log10 adjusted p-value (g:SCS correction, details in Methods) of the genes associated with spatial PC values.

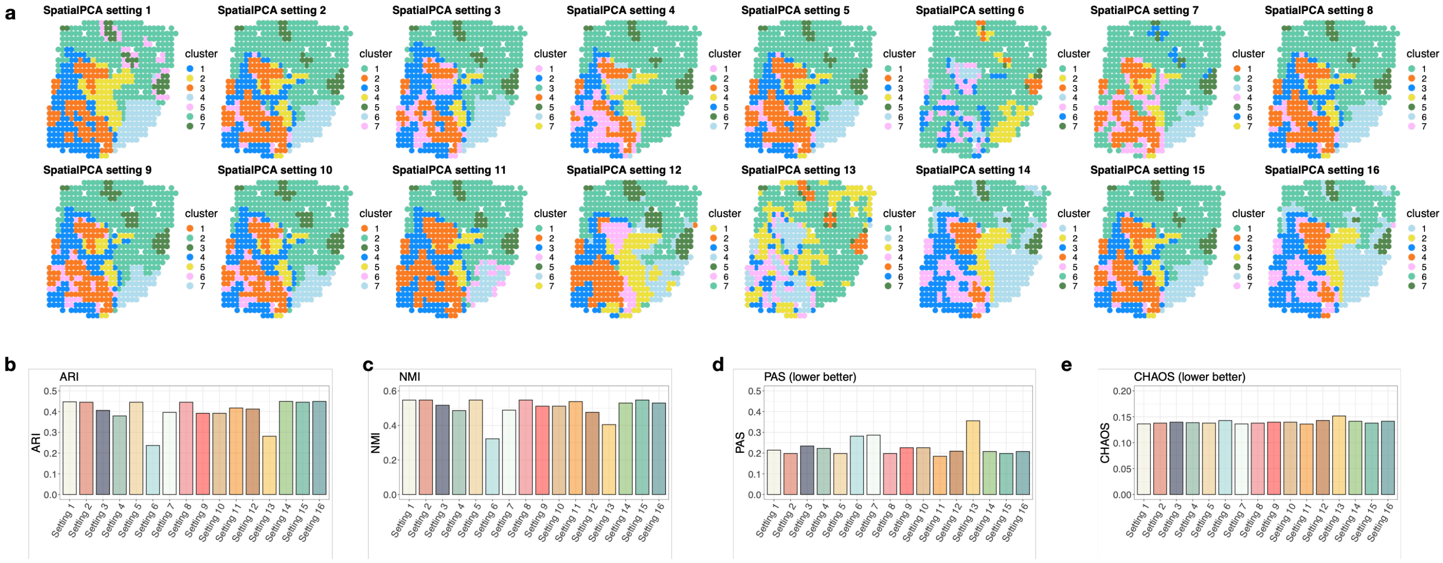

Supplementary Figure 34. Sensitivity analyses for the ST data. **a.** Clustering results from SpatialPCA are shown for different analytic settings. Setting 1: using top 10 Spatial PCs. Setting 2: using top 20 Spatial PCs. Setting 3: using top 30 Spatial PCs. Setting 4: using top 50 Spatial PCs. Setting 5: using all spatially variable genes detected by SPARK. Setting 6: using all highly variable genes detected by Seurat. Setting 7: using all spatially variable genes detected by SPARK-X. Setting 8: using Gaussian kernel. Setting 9: using Cauchy kernel. Setting 10: using quadratic kernel. Setting 11: controlling for cell types when selecting SVGs in SPARK-X. Setting 12: controlling for cell types in SpatialPCA. Setting 13: controlling for cell types by regressing them out from the input gene expression and take the residuals. Setting 14: gene expression normalized through SCTransform normalization. Setting 15: gene expression normalized through log normalization. Setting 16: taking the histology information as a third dimension in location matrix. **b.** Clustering accuracy as measured by adjusted Rand index (ARI, the higher the better) for different settings. **c.** Clustering accuracy as measured by normalized mutual information (NMI, the higher the better) for different settings. **d.** Spatial continuity of the inferred clusters as measured by percentage of abnormal spots (PAS, the lower the better) for different settings. **e.** Spatial continuity of the inferred clusters as measured by spatial chaos score (CHAOS, the lower the better) for different settings.

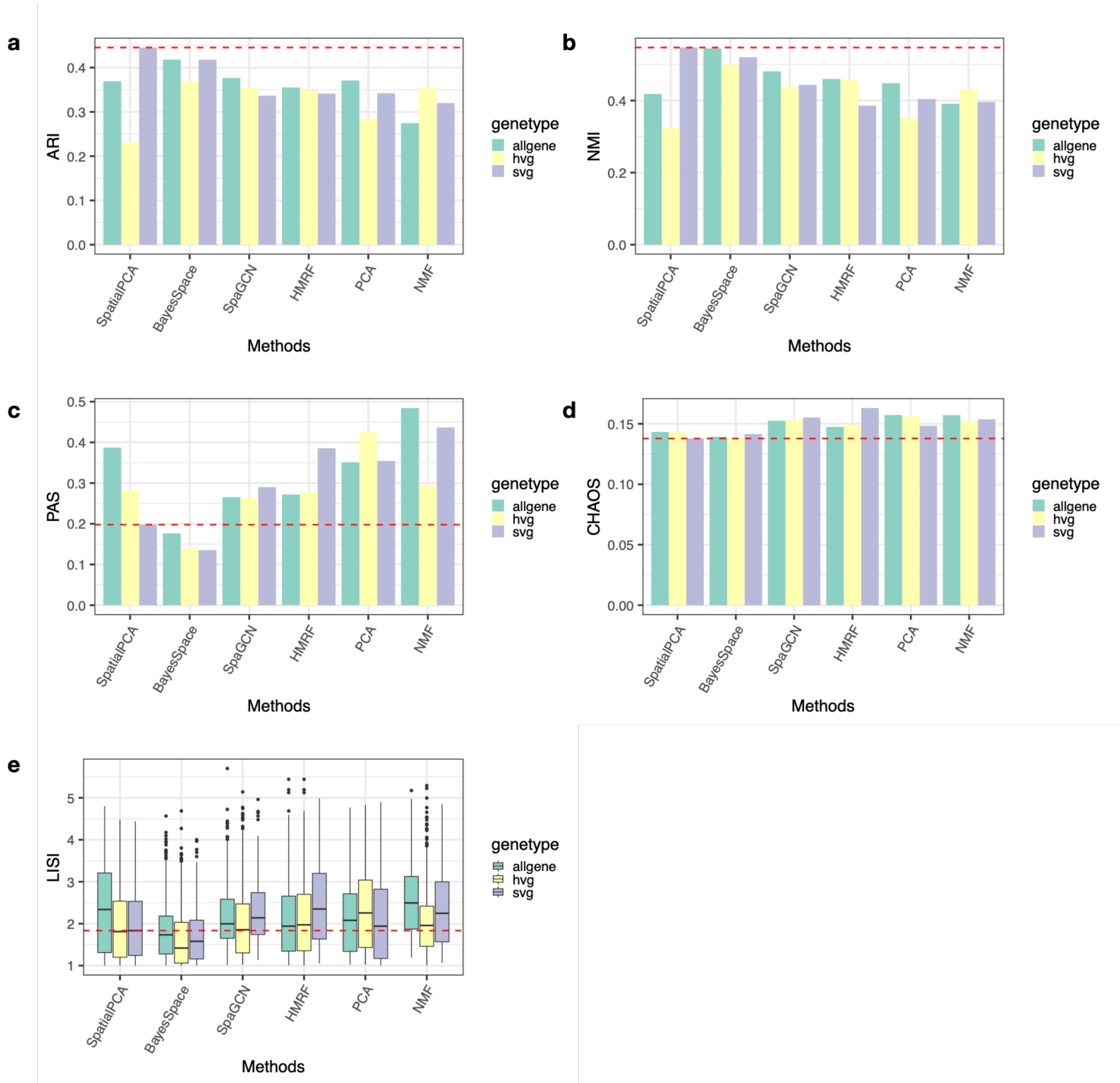

Supplementary Figure 35. Clustering results obtained using different methods for the ST data. a. Clustering accuracy measured by adjusted Rand index (ARI, the higher the better). In dimension reduction methods (SpatialPCA, PCA, and NMF), clustering was performed based on the inferred low-dimensional components. For spatial domain clustering methods (BayesSpace, SpaGCN and HMRF), clustering was performed based the default settings. All methods are performed under three different analytic settings: either using SVGs, HVGs, or all genes. **b-e**. Clustering accuracy measured by normalized mutual information (NMI, the higher the better), and spatial continuity measured by percentage of abnormal spots (PAS, the lower the better), spatial chaos score (CHAOS, the lower the better) and local inverse Simpson's index (LISI, the lower the better) for different methods on 607 spots. In the boxplot in **e**, the center line, box limits and whiskers denote the median, upper and lower quartiles, and 1.5× interquartile range, respectively.

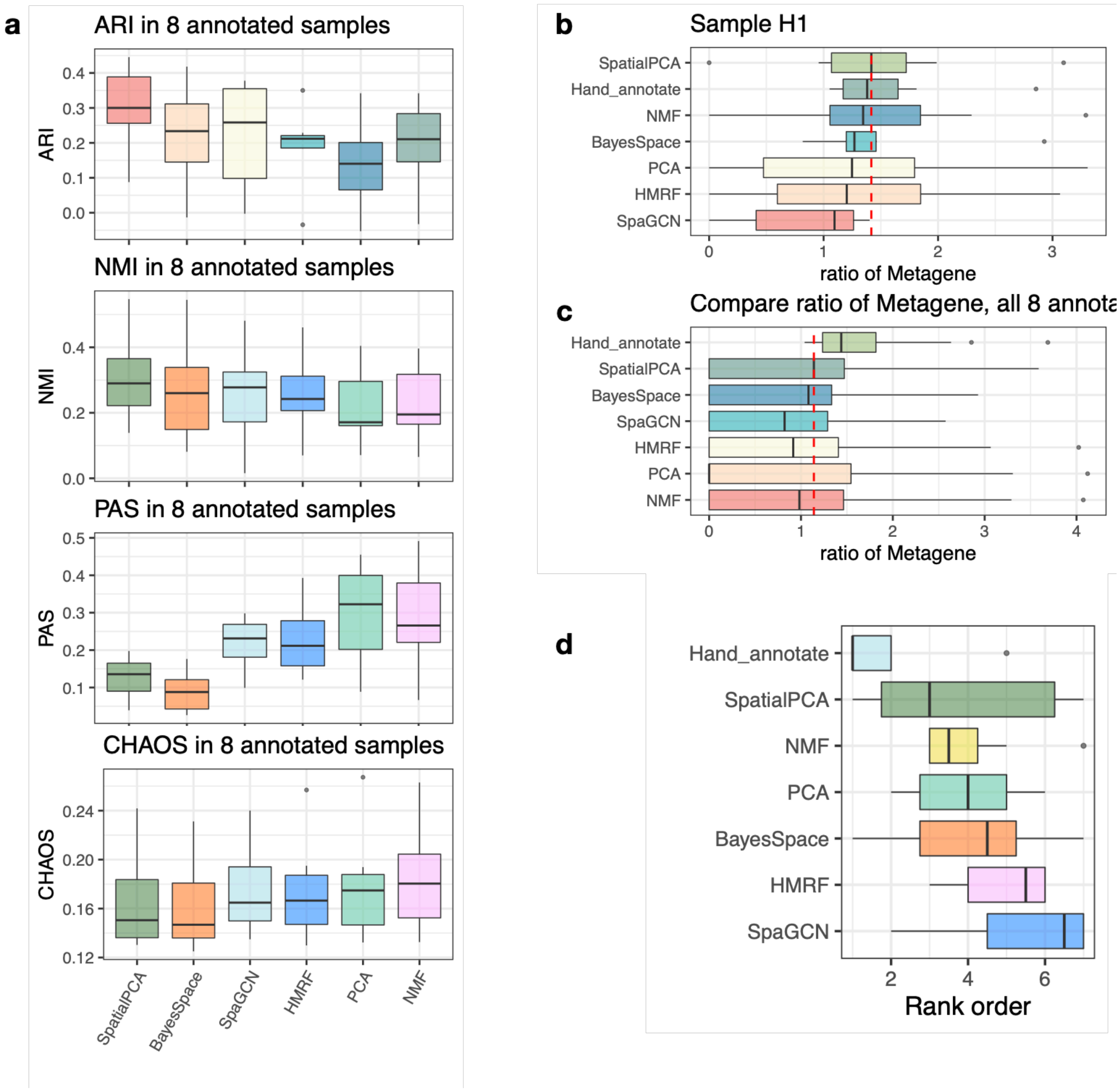

Supplementary Figure 36. Clustering results in the ST data. a. Clustering accuracy of different methods are measured by adjusted Rand index (ARI, the higher the better) and normalized mutual information (NMI, the higher the better), while the spatial continuity of the clusters from different methods are measured by percentage of abnormal spots (PAS, the lower the better) and spatial chaos score (CHAOS, the lower the better). Results are shown for all 8 annotated samples in the form of boxplots. **b.** The enrichment scores for the seven domain-specific metagenes from each method is shown in the form of a boxplot in the H1 sample. Higher score indicates better retrieving of the fine-grained transcriptomic details of the detected spatial regions. Results are shown for all 8 annotated samples in the form of boxplots. **c.** The metagene enrichment score for each method is shown across all 8 annotated samples in the form of a boxplot. **d.** Ranking of different methods in terms of the metagene enrichment score across all 8 annotated tissue samples. In the boxplots in **a**-**d**, the center line, box limits and whiskers denote the median, upper and lower quartiles, and 1.5× interquartile range, respectively.

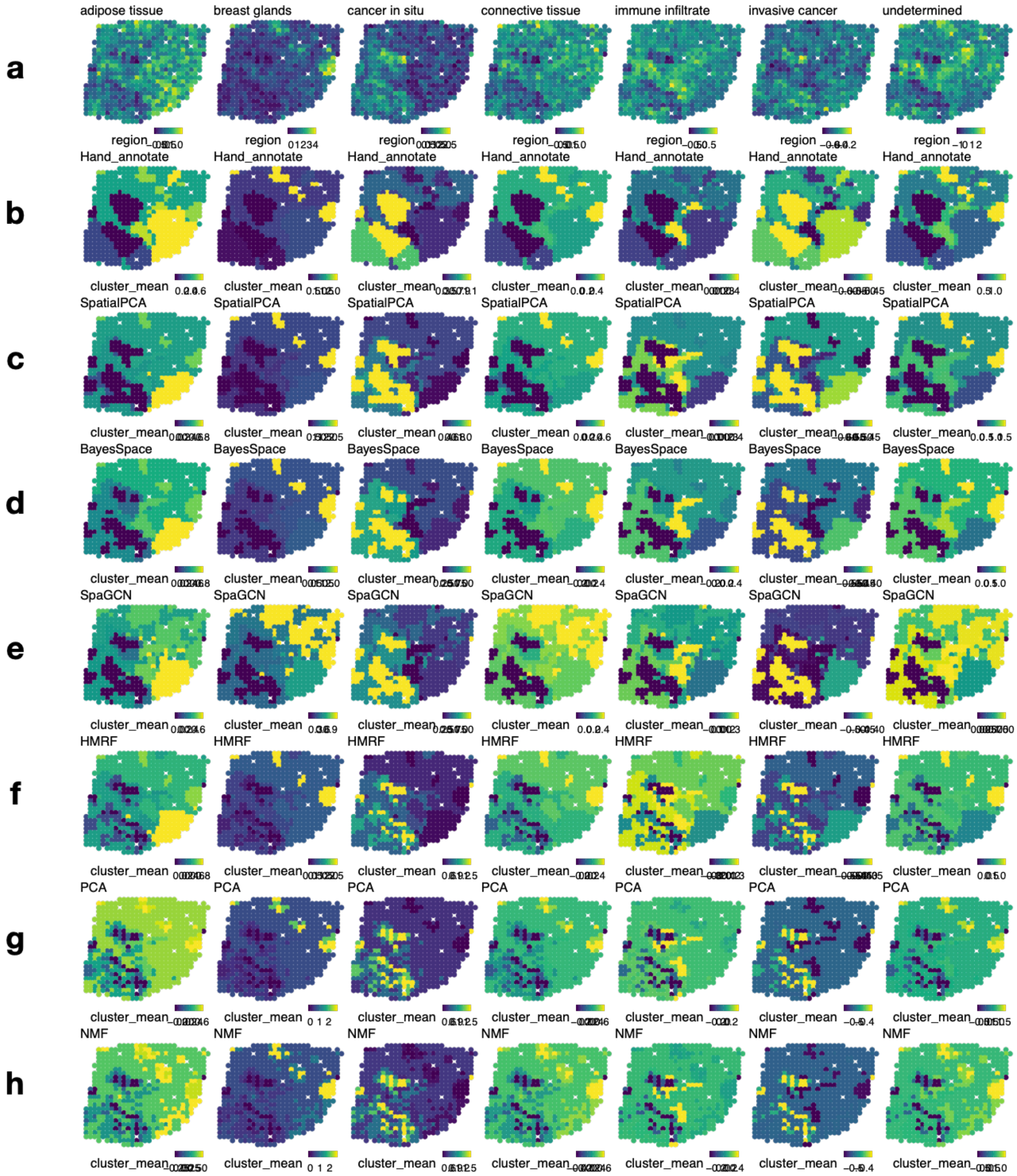

Supplementary Figure 37. Metagene expression in the ST data. a. The expression of eight domain specific metagenes is displayed in the H1 sample. The eight metagenes are specifically expressed in eight tissue domains that include adipose tissue, breast glands, cancer in situ, connective tissue, immune infiltrate, invasive cancer, and undetermined. **b-h**. The average expression of each metagene within each spatial domain detected by different methods are displayed in the H1 sample. Different methods include pathologist annotation (**b**), SpatialPCA (**c**), BayesSpace (**d**), SpaGCN (**e**), HMRF (**f**), PCA (**g**), or NMF (**h**).

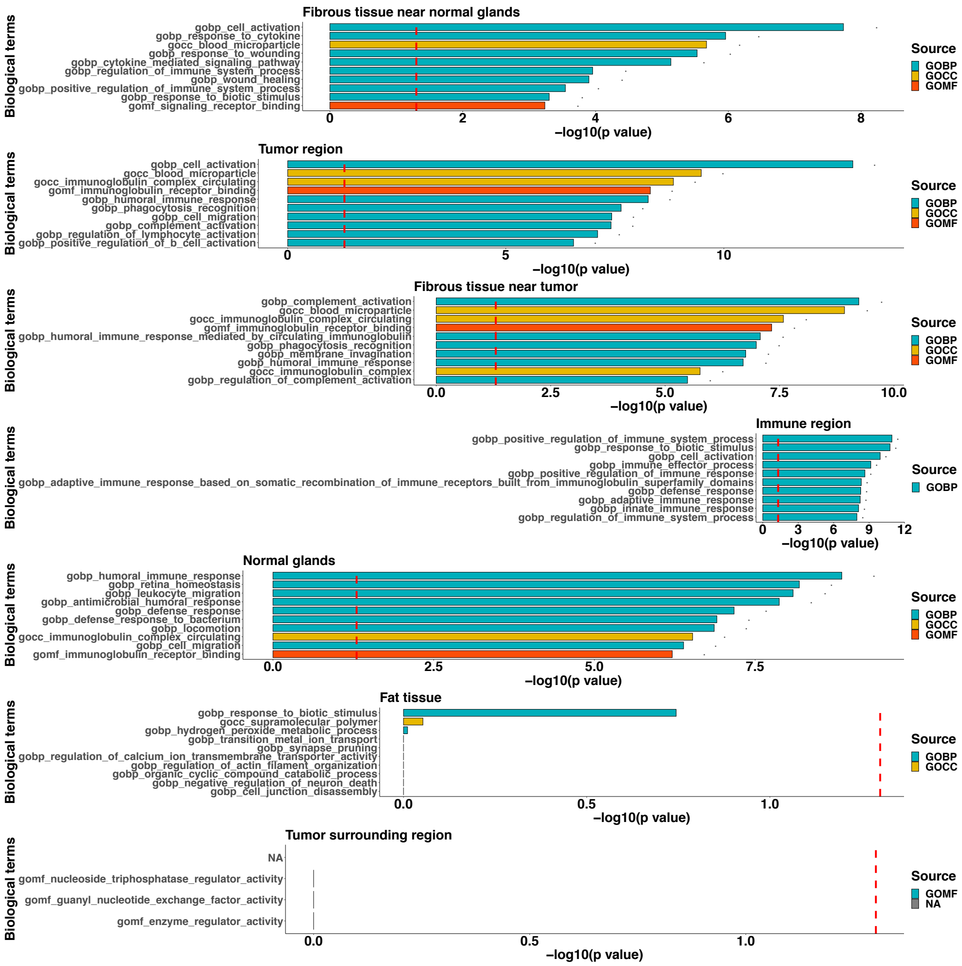

Supplementary Figure 38. Gene set enrichment analysis on the region-specific genes in the ST tumor data. The top 10 enriched gene sets are shown for each of the seven detected tissue regions. Color represents different data sources for annotating the gene sets. The enrichment is given as -log10 adjusted p-value (g:SCS correction, details in Methods) of the region specific genes.

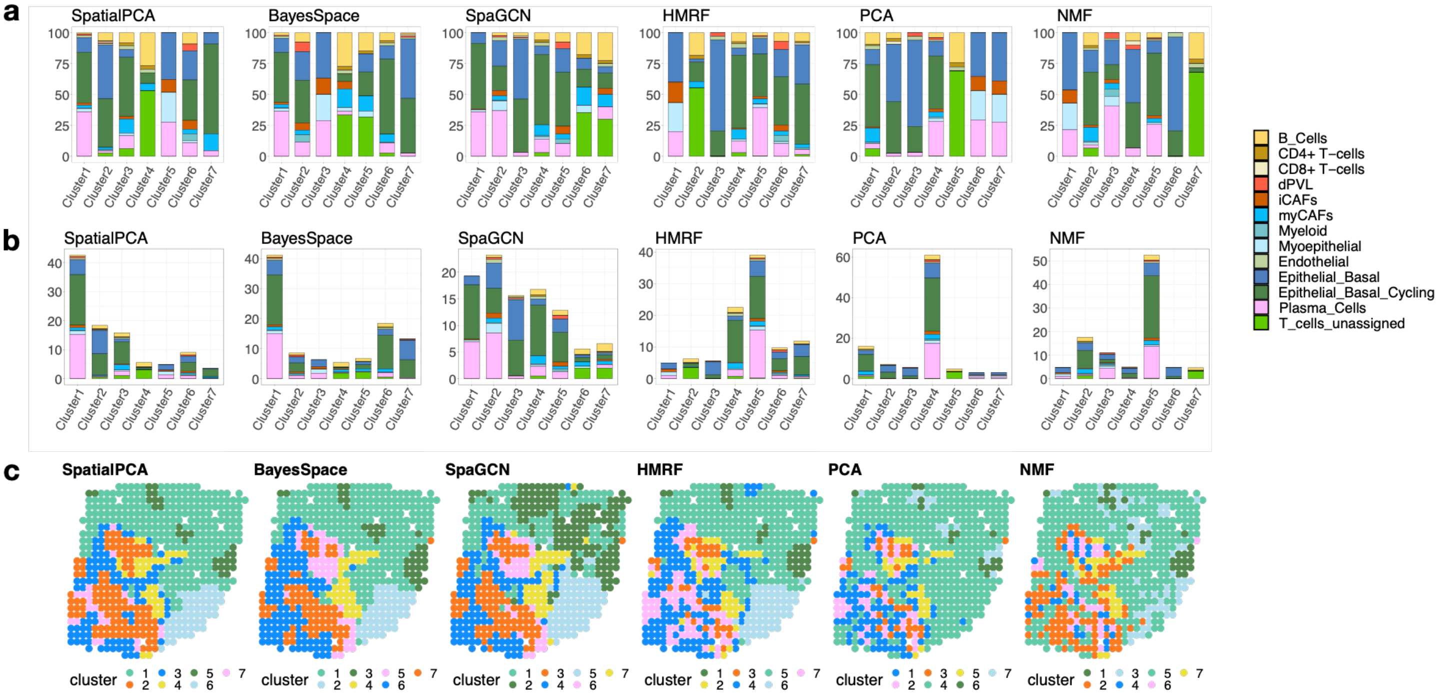

Supplementary Figure 39. Comparison of the cell type composition of the spatial domains detected by different methods in the ST tumor data. The percentage of cell types annotated (y-axis) is shown on each tissue domain (x-axis) detected by different methods. Examined methods include SpatialPCA, BayesSpace, SpaGCN, HMRF, PCA, and NMF. **a**. Results are scaled with respect to each spatial domain, such that the summation of the cell type percentages in each domain is 100%. **b**. Results are scaled with respect to the cell types, such that the summation of all cell types across all tissue regions is 100%. **c**. Clustering results in each method. The clustering labels correspond to the x-axis in the left and middle panel. The clustering labels correspond to the x-axis in the left and middle panel. The reference scRNA-seq data for ST data that contain immune cell types and malignant cell types is collected on breast cancer via the InDrop platform.

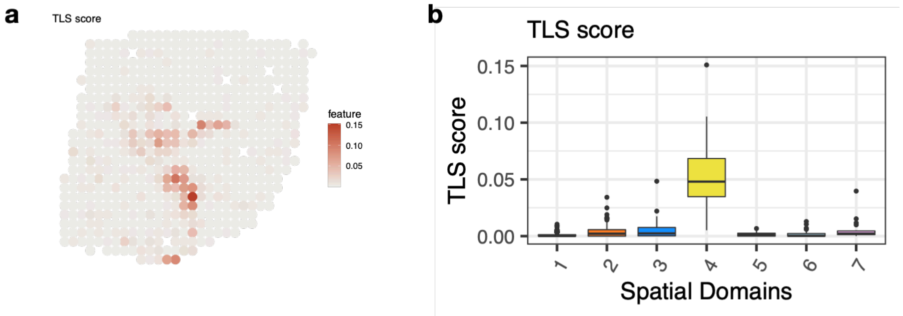

Supplementary Figure 40. TLS score in ST tumor data. **a.** Visualization of TLS score in the H1 sample. **b.** The distribution of TLS scores in different spatial domains detected by SpatialPCA across all 607 spots. Cluster 4 is the TLS region detected by SpatialPCA. In the boxplot, the center line, box limits and whiskers denote the median, upper and lower quartiles, and 1.5× interquartile range, respectively.

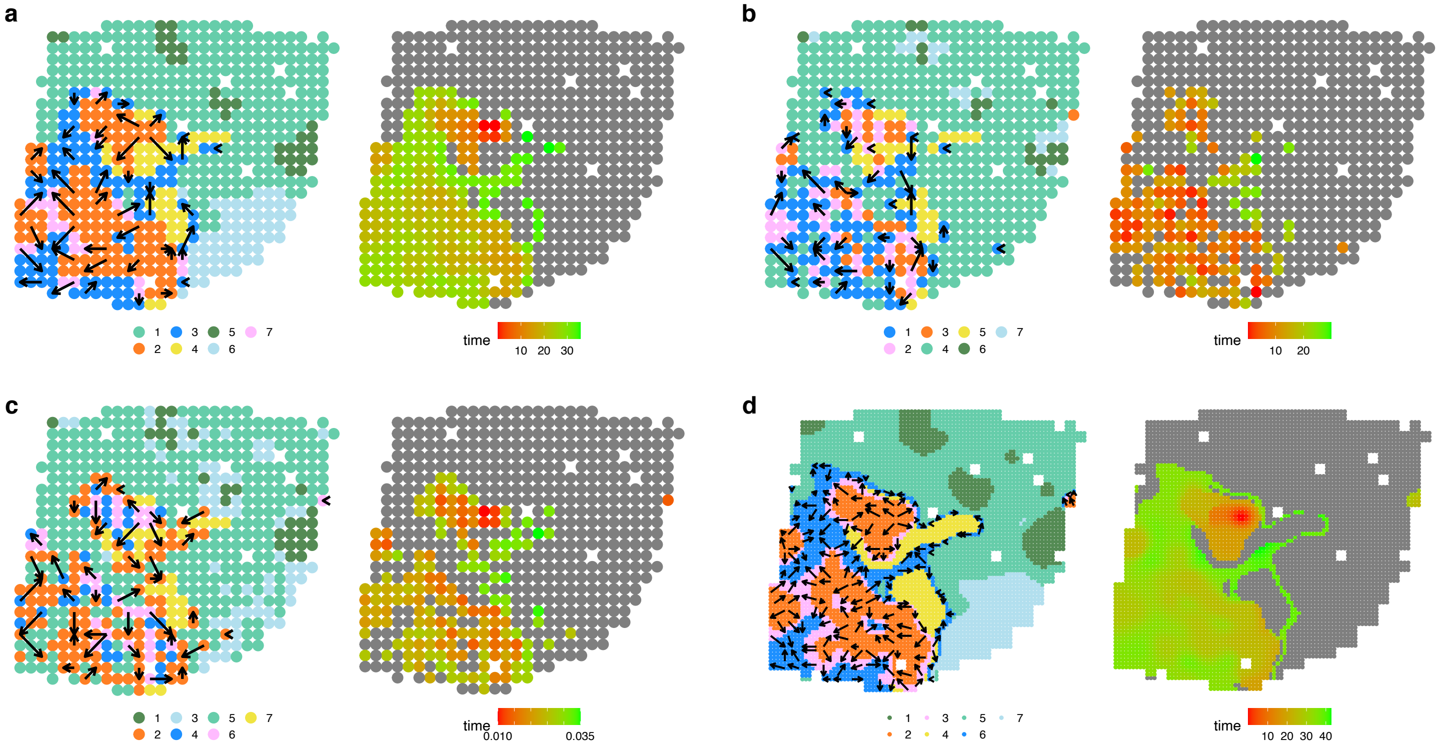

Supplementary Figure 41. Spatial trajectory inference results in tumor and surrounding regions in the ST tumor data. **a.** Visualization of the trajectory inferred by SpatialPCA in the original data. Left: Arrows point from tissue locations with low pseudo-time to tissue locations with high pseudo-time. Color represents different tissue regions. Right: Visualization of pseudotime inferred from spatial PCs in SpatialPCA. **b.** Visualization of the trajectory inferred by PCA. **c.** Visualization of the trajectory inferred by NMF. **d.** Visualizaton of the three trajectories inferred by SpatialPCA on the SpatialPCA constructed high-resolution spatial map. In all panels, arrows point from tissue locations with low pseudo-time to tissue locations with high pseudo-time. Color represents different tissue regions.

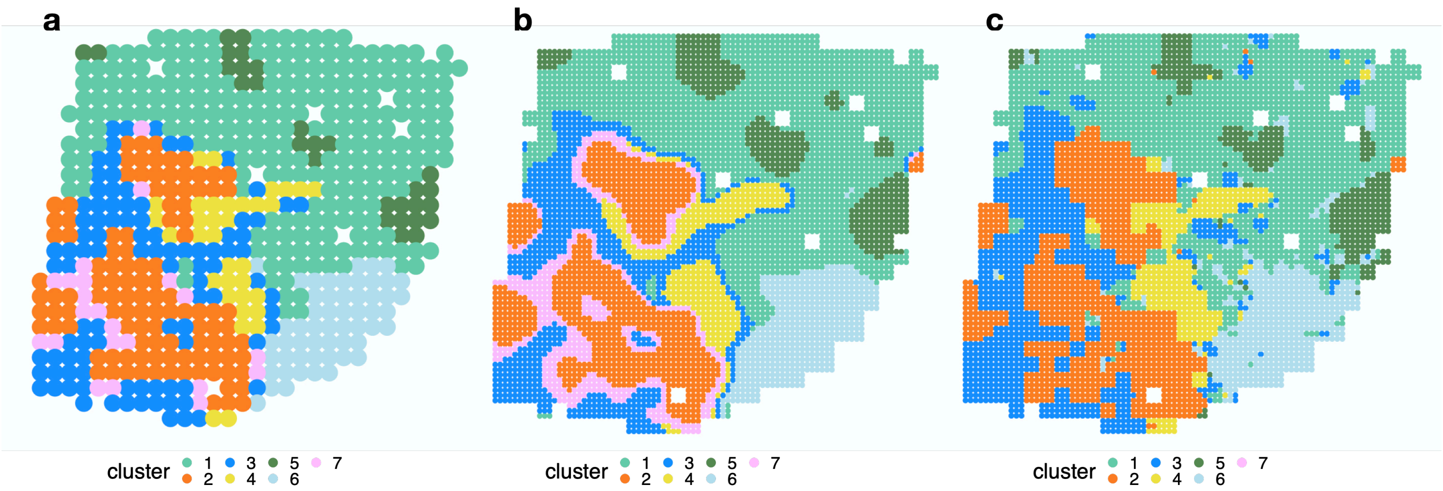

Supplementary Figure 42. Visualization of high-resolution spatial map prediction in the ST data. a. The spatial domain clustering results using SpatialPCA based on the original resolution. **b.** Visualization of high-resolution spatial map prediction in SpatialPCA with default setting. **c.** Visualization of high-resolution spatial map prediction in BayesSpace with default setting.

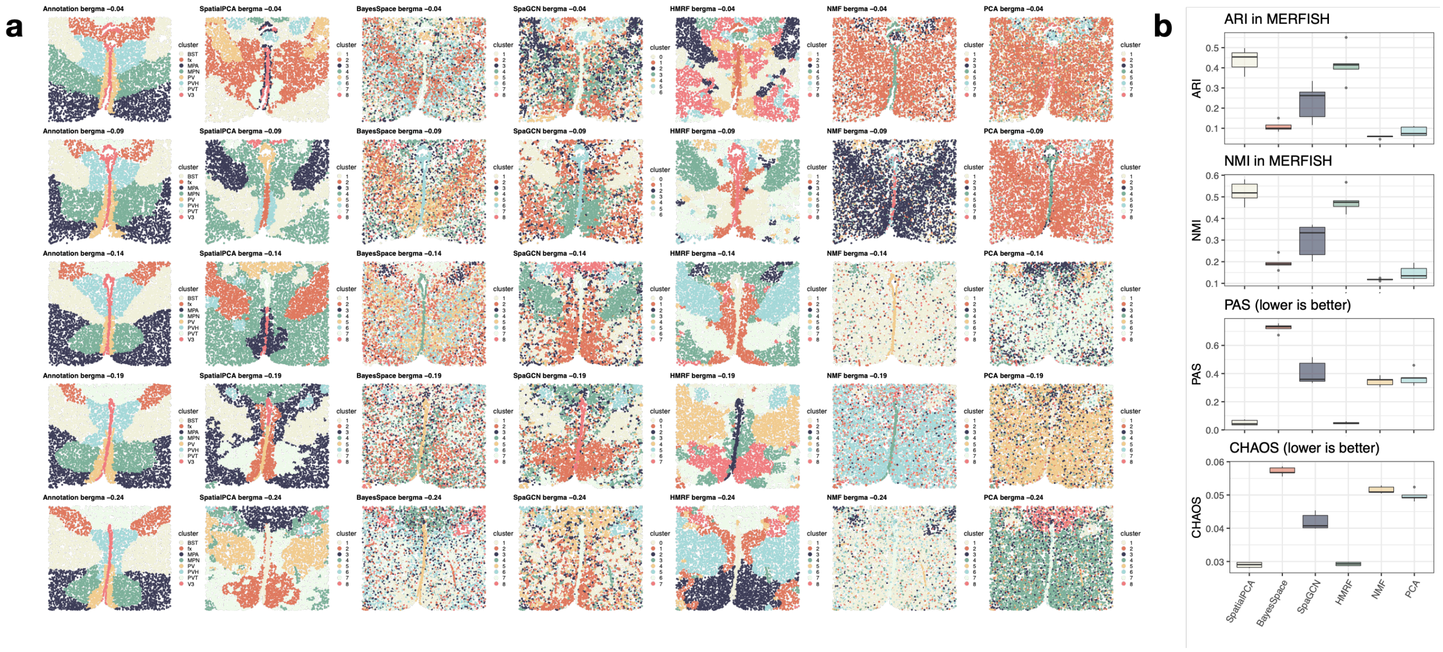

Supplementary Figure 43. Comparison of the spatial domains clustering results by different methods in the MERFISH data. **a.** Clustering results for different tissue sections (rows) by different methods (columns). The ground truth annotation (1st column) is manually annotated according to the cell type and marker gene expression in[3]. The examined methods include SpatialPCA (2nd column), BayesSpace (3rd column), SpaGCN (4th column), HMRF (5th column), NMF (6th column), and PCA (7th column). The MERFISH sample[4] is measured on 155 genes and an average of 5,663 cells and includes five tissue sections collected at bregma values -0.04, -0.09, -0.14, -0.19, and -0.24. **b.** Clustering results measured by adjusted Rand index (ARI, the higher the better), normalized mutual information (NMI, the higher the better), spatial chaos score (CHAOS, the lower the better), and percentage of abnormal spots (PAS, the lower the better) for different methods with their default settings across 5 sections. Clustering results of PCA and NMF are obtained with SVGs. The MERFISH data[4] is collected on the preoptic region of the mouse hypothalamus using the multiplexed error-robust fluorescent *in situ* hybridization-based technology. In the boxplots in **b**, the center line, box limits and whiskers denote the median, upper and lower quartiles, and 1.5× interquartile range, respectively.

Supplementary Tables

Supplementary Table 1. Median ARI values of simulations in four scenarios at single cell level. (Default settings in each method are shaded in green color.)

Methods	scenario1	scenario2	scenario3	scenario4
**SpatialPCA SVGs**	0.942	0.877	0.931	0.439
SpatialPCA HVGs	0.901	0.864	0.882	0.504
SpatialPCA all genes	0.893	0.851	0.877	0.518
BayesSpace SVGs	0.312	0.08	0.205	0.005
**BayesSpace HVGs**	0.367	0.225	0.286	0.075
BayesSpace all genes	0.363	0.167	0.215	0.064
SpaGCN SVGs	0.635	0.264	0.411	0.091
SpaGCN HVGs	0.626	0.278	0.408	0.091
**SpaGCN all genes**	0.625	0.277	0.412	0.138
HMRF_SVGs	0.9	0.341	0.845	0.01
HMRF HVGs	0.83	0.285	0.718	0
**HMRF all genes**	0.773	0.279	0.671	0.002
**NMF SVGs**	0.365	0.119	0.285	0.071
NMF HVGs	0.343	0.21	0.27	0.07
NMF all genes	0.339	0.206	0.264	0
**PCA SVGs**	0.362	0.109	0.264	0.075
PCA HVGs	0.363	0.221	0.283	0.075
PCA all genes	0.367	0.225	0.286	0.439

Supplementary Table 2. Median ARI values of simulations in four scenarios at spot level (spot diameter is 90um).

Methods	**scenario1**	**scenario2**	**scenario3**	**scenario4**
SpatialPCA	0.955	0.953	0.969	0.962
BayesSpace	0.759	0.759	0.864	0.763
SpaGCN	0.719	0.925	0.966	0.874
HMRF	0.82	0.806	0.898	0.843
NMF	0.573	0.57	0.749	0.598
PCA	0.564	0.543	0.757	0.592

Supplementary Table 3. Median NMI values of simulations in four scenarios at spot level (spot diameter is 90um).

Methods	scenario1	scenario2	scenario3	scenario4
SpatialPCA	0.927	0.925	0.946	0.933
BayesSpace	0.697	0.672	0.792	0.733
SpaGCN	0.709	0.876	0.942	0.834
HMRF	0.74	0.726	0.836	0.763

NMF	0.515	0.484	0.66	0.581
PCA	0.535	0.465	0.674	0.622

Supplementary Table 4. Median CHAOS values of simulations in four scenarios at spot level (spot diameter is 90um).

Methods	scenario1	scenario2	scenario3	scenario4
SpatialPCA	0.042	0.042	0.042	0.042
BayesSpace	0.048	0.05	0.049	0.045
SpaGCN	0.045	0.044	0.043	0.044
HMRF	0.047	0.048	0.047	0.047
NMF	0.051	0.053	0.051	0.049
PCA	0.05	0.053	0.05	0.047

Supplementary Table 5. Median PAS values of simulations in four scenarios at spot level (spot diameter is 90um).

Methods	scenario1	scenario2	scenario3	scenario4
SpatialPCA	0.007	0.007	0.005	0.009
BayesSpace	0.109	0.109	0.062	0.112
SpaGCN	0.101	0.03	0.013	0.051
HMRF	0.07	0.075	0.039	0.063
NMF	0.211	0.209	0.116	0.207
PCA	0.214	0.226	0.111	0.218

Supplementary Table 6. Number of genes associated with spatial PC values detected. The results are shown in DLPFC data sample 151676 (first column), Slide-seq data (second column), Slide-seq V2 data (third column), and ST tumor data (fourth column).

Spatial PCs	DLPFC	Slide-seq	Slide-seqV2	ST
**SpatialPC1**	929	263	1288	133
**SpatialPC2**	395	126	1342	32
**SpatialPC3**	287	126	833	40
**SpatialPC4**	165	95	1034	19
**SpatialPC5**	64	112	799	13
**SpatialPC6**	53	77	470	18
**SpatialPC7**	42	28	668	11
**SpatialPC8**	51	48	564	9
**SpatialPC9**	18	46	431	10
**SpatialPC10**	18	57	554	8
**SpatialPC11**	12	58	258	8
**SpatialPC12**	9	44	465	2
**SpatialPC13**	10	38	475	8
**SpatialPC14**	8	26	247	6
**SpatialPC15**	9	36	284	10
**SpatialPC16**	2	35	387	2
**SpatialPC17**	3	57	293	3
**SpatialPC18**	5	37	303	8
**SpatialPC19**	5	45	202	3
**SpatialPC20**	6	36	296	1

Supplementary Table 7. Median ARI values of 12 samples in the DLPFC data. Default settings in each method are shaded in green color. The default setting in stLearn is to use marker genes, median ARI=0.470.

Methods/Gene type	SVGs	HVGs	All genes
**SpatialPCA**	**0.542**	0.450	0.303
**BayesSpace**	0.479	**0.438**	0.449
**SpaGCN**	0.190	0.208	**0.443**
**HMRF**	0.310	0.292	0.304
**stLearn**	0.311	0.341	0.345
**PCA**	0.358	0.305	0.345
**NMF**	0.262	0.123	0.263

Supplementary Table 8. Number of region-specific genes in each of the seven spatial domains detected using SpatialPCA in the DLPFC data (sample 151676).

Spatial domain name	Number of region-specific genes
Cluster 1	79
Cluster 2	777
Cluster 3	124
Cluster 4	66
Cluster 5	127
Cluster 6	153
Cluster 7	203

Supplementary Table 9. Number of pseudo-time associated genes in the DLPFC data (sample 151676), cortical layers in Slide-seq V2 data, and tumor regions in ST tumor data.

Dataset	Trajectory number	Number of pseudo-time associated genes
DLPFC	1	716
Slide-seq V2 cortical layers	1	883
ST tumor	1	40

Supplementary Table 10. Number of region-specific genes in each of the eight spatial domains detected using SpatialPCA in the Slide-seq data.

Spatial domain name	Number of region-specific genes
Choroid plexus	3
White matter	9
GCL middle layer	7
GCL inner sublayer	2
Cerebellum nuclei	4
Molecular layer	9
GCL outer layer	3
Purkinje layer	14

Supplementary Table 11. Number of region-specific genes in each of the 14 spatial domains detected using SpatialPCA in the Slide-seq V2 data.

Spatial domain name	Number of region-specific genes	Spatial domain name	Number of region-specific genes
CA1	72	Dentate gyrus	61
Third ventricle	34	CA3	114
Layer 6	20	Corpus callosum	66
Hippocampus (slm)	39	Thalamus subregion1	24
Layer 4	32	Thalamus subregion 3	38
Hippocampus (so/sr)	32	Thalamus subregion 2	16
Layer 5	14	Hippocampus (so)	17

Supplementary Table 12. Number of region-specific genes detected in each of the seven spatial domains detected using SpatialPCA in the ST tumor data.

Spatial domain name	Number of region-specific genes
Fibrous tissue near normal glands	132
Fat tissue	25
Immune region	72
Tumor surrounding region	0
Tumor region	240
Fibrous tissue near tumor	14
Normal glands	53

Supplementary Table 13. Computation time and peak memory usage for SpatialPCA, BayesSpace, SpaGCN, HMRF, PCA, and NMF in the three real data applications. Computing time is recorded in dataset using a single thread on an Intel(R) Xeon(R) Gold 6138 CPU @ 2.00GHz processor.

	**DLPFC data sample 151676 (n=3460)**	**Slide-seq data (n=25,551)**	**Slide-seq V2 data (n=53,208)**	**ST data (n=613)**
**SpatialPCA** Time / Memory	6min / 2212Mb	23min / 11Gb	6.1 hours / 70Gb	11s / 157 Mb, high resolution: 1s (322Mb)
**BayesSpace** Time / Memory	27min / 10.5Gb	4.1 hours / 40Gb	>100Gb, did not run	85s / 713Mb, high resolution: 22min / 1026Mb
**SpaGCN** Time / Memory	28s / 1095Mb	6min / 7Gb	1.2 hours / 45Gb	11s / 89Mb
**stLearn** Time / Memory	5min / 201Mb	-	-	-
**HMRF** Time / Memory	3min/3873Mb	58min/18Gb	>100Gb, did not run	1min/326Mb
**PCA** Time / Memory	3s / 542Mb	8s / 882Mb	2.8min / 3.8Gb	1s / 21Mb
**NMF** Time / Memory	4s / 172Mb	6s / 806Mb	19s / 1070Mb	1s / 30Mb

Supplementary References:

1 Kokiopoulou, E., Chen, J. & Saad, Y. Trace optimization and eigenproblems in dimension reduction methods. *Numerical Linear Algebra with Applications* **18**, 565-602, doi:10.1002/nla.743 (2011).

2 Saad, Y. Numerical Methods for Large Eigenvalue Problems Preface to the Classics Edition. *Numerical Methods for Large Eigenvalue Problems, Revised Edition* **66**, Xiii-+, doi:Book_Doi 10.1137/1.9781611970739 (2011).

3 Li, Z. & Zhou, X. BASS: multi-scale and multi-sample analysis enables accurate cell type clustering and spatial domain detection in spatial transcriptomic studies. *Genome Biology* **23**, 168, doi:10.1186/s13059-022-02734-7 (2022).

4 Moffitt, J. R. *et al.* Molecular, spatial, and functional single-cell profiling of the hypothalamic preoptic region. *Science* **362**, doi:10.1126/science.aau5324 (2018).
