# Supplementary material for: Spatially aware dimension reduction for spatial transcriptomics
Source: Nat Commun. 2022 Nov 23;13:7203. doi: 10.1038/s41467-022-34879-1 (PMC9684472; doi:10.1038/s41467-022-34879-1)
Supplement: Supplementary file 3 — Reporting Summary [file 41467_2022_34879_MOESM3_ESM.pdf]

nature portfolio

Corresponding author(s): Xiang Zhou

Last updated by author(s): Nov 3, 2022

Reporting Summary

Nature Portfolio wishes to improve the reproducibility of the work that we publish. This form provides structure for consistency and transparency in reporting. For further information on Nature Portfolio policies, see our Editorial Policies and the Editorial Policy Checklist.

Statistics

For all statistical analyses, confirm that the following items are present in the figure legend, table legend, main text, or Methods section.

n/a	Confirmed	
☐	☒	The exact sample size (n) for each experimental group/condition, given as a discrete number and unit of measurement
☐	☒	A statement on whether measurements were taken from distinct samples or whether the same sample was measured repeatedly
☐	☒	The statistical test(s) used AND whether they are one- or two-sided<br>*Only common tests should be described solely by name; describe more complex techniques in the Methods section.*
☒	☐	A description of all covariates tested
☐	☒	A description of any assumptions or corrections, such as tests of normality and adjustment for multiple comparisons
☒	☐	A full description of the statistical parameters including central tendency (e.g. means) or other basic estimates (e.g. regression coefficient) AND variation (e.g. standard deviation) or associated estimates of uncertainty (e.g. confidence intervals)
☒	☐	For null hypothesis testing, the test statistic (e.g. F, t, r) with confidence intervals, effect sizes, degrees of freedom and P value noted<br>*Give P values as exact values whenever suitable.*
☐	☒	For Bayesian analysis, information on the choice of priors and Markov chain Monte Carlo settings
☒	☐	For hierarchical and complex designs, identification of the appropriate level for tests and full reporting of outcomes
☐	☒	Estimates of effect sizes (e.g. Cohen's d, Pearson's r), indicating how they were calculated

Our web collection on statistics for biologists contains articles on many of the points above.

Software and code

Policy information about availability of computer code

Data collection	No software was used for data collection.
Data analysis	We used the newly developed R package SpatialPCA for data analysis. SpatialPCA is described in the Methods section and deposited at Github (https://github.com/shangll123/SpatialPCA, R version 1.3.0). The source code is released under the GNU General Public License version 3 (GPL >=3). Example codes for using SpatialPCA are publicly available at http://lulushang.org/SpatialPCA_Tutorial/index.html. All analysis codes for reproducing the results of the present study are publicly available at https://github.com/shangll123/SpatialPCA_analysis_codes.<br><br>We used the following software packages for comparative analysis:<br>stLearn [https://stlearn.readthedocs.io/en/latest/] (Python package v0.3.1)<br>SpaGCN [https://github.com/jianhuupenn/SpaGCN] (R package v1.2.0)<br>BayesSpace [https://github.com/edward130603/BayesSpace] (R package v1.6.0)<br>Giotto [https://rubd.github.io/Giotto_site/] (R package v1.0.3)<br><br>in addition, we also used the following software packages for data analysis:<br>Seurat [https://github.com/satijalab/seurat] (R package v4.0.5)<br>RCTD [https://github.com/dmcable/spacexr] (R package V1.1.0)

For manuscripts utilizing custom algorithms or software that are central to the research but not yet described in published literature, software must be made available to editors and reviewers. We strongly encourage code deposition in a community repository (e.g. GitHub). See the Nature Portfolio guidelines for submitting code & software for further information.

Data

Policy information about availability of data

All manuscripts must include a data availability statement. This statement should provide the following information, where applicable:

- Accession codes, unique identifiers, or web links for publicly available datasets
- A description of any restrictions on data availability
- For clinical datasets or third party data, please ensure that the statement adheres to our policy

This study made use of publicly available datasets. The human DLPFC samples are available at http://spatial.libd.org/spatialLIBD/. ST data is available at https://github.com/almaan/her2st. Slide-Seq data is available at Broad Institute's single-cell repository (https://singlecell.broadinstitute.org/single_cell/) with ID SCP354. Slide-seq V2 data is available at Broad Institute's single-cell repository (https://singlecell.broadinstitute.org/single_cell/) with ID SCP815. The scRNA-seq reference data used in this study are all publicly available, including GSE104276 [https://www.ncbi.nlm.nih.gov/geo/query/acc.cgi?acc=GSE104276] for human prefrontal cortex data; http://dropviz.org for mouse cerebellum and hippocampus data; and GSE114725 [https://www.ncbi.nlm.nih.gov/geo/query/acc.cgi?acc=GSE114725] for human breast tumor data.

The databases we used include:
Molecular Signatures Database (https://www.gsea-msigdb.org/gsea/msigdb)
Mouse Genome Database (http://www.informatics.jax.org)
Allen Brain Atlas (https://www.brain-map.org)

Human research participants

Policy information about studies involving human research participants and Sex and Gender in Research.

Reporting on sex and gender	N/A
Population characteristics	N/A
Recruitment	N/A
Ethics oversight	N/A

Note that full information on the approval of the study protocol must also be provided in the manuscript.

Field-specific reporting

Please select the one below that is the best fit for your research. If you are not sure, read the appropriate sections before making your selection.

☒ Life sciences ☐ Behavioural & social sciences ☐ Ecological, evolutionary & environmental sciences

For a reference copy of the document with all sections, see nature.com/documents/nr-reporting-summary-flat.pdf

Life sciences study design

All studies must disclose on these points even when the disclosure is negative.

Sample size	No sample size calculation was performed. SpatialPCA was evaluated across four publicly available spatially resolved transcriptomics datasets in real data applications, including 12 sample sections in the DLPFC dataset; one mouse cerebellum data from Slide-seq dataset; one mouse hippocampus data from Slide-seq V2 dataset; and one human breast cancer data from 10X ST dataset.
Data exclusions	Following standard practice, we retained genes with non-zero expression level on at least 20 locations and retained locations with non-zero expression for at least 20 genes for analysis, in order to avoid false positives.
Replication	We did not perform replication. Instead, we cross-validate the findings of the present study by comparing to other published molecular biology results.
Randomization	Randomization is not relevant to this study because each sample or slide was analyzed separately.
Blinding	Blinding is not relevant to our study because we don't compare any case/control groups.

Reporting for specific materials, systems and methods

We require information from authors about some types of materials, experimental systems and methods used in many studies. Here, indicate whether each material, system or method listed is relevant to your study. If you are not sure if a list item applies to your research, read the appropriate section before selecting a response.

Materials & experimental systems

n/a	Involved in the study
☒	☐ Antibodies
☒	☐ Eukaryotic cell lines
☒	☐ Palaeontology and archaeology
☒	☐ Animals and other organisms
☒	☐ Clinical data
☒	☐ Dual use research of concern

Methods

n/a	Involved in the study
☒	☐ ChIP-seq
☒	☐ Flow cytometry
☒	☐ MRI-based neuroimaging
